# HPLC-PDA-ESI-HRMS-Based Profiling of Secondary Metabolites of *Rindera graeca* Anatomical and Hairy Roots Treated with Drought and Cold Stress

**DOI:** 10.3390/cells11060931

**Published:** 2022-03-08

**Authors:** Marcin Robert Naliwajski, Beata Wileńska, Aleksandra Misicka, Agnieszka Pietrosiuk, Katarzyna Sykłowska-Baranek

**Affiliations:** 1 Department of Plant Physiology and Biochemistry, Faculty of Biology and Environmental Protection, University of Lodz, 12/16 Banacha St., 90-237 Lodz, Poland; marcin.naliwajski@biol.uni.lodz.pl; 2 Faculty of Chemistry, University of Warsaw, 1 Pasteura St., 02-093 Warsaw, Poland; misicka@chem.uw.edu.pl; 3 Biological and Chemical Research Centre, 101 Żwirki i Wigury St., 02-097 Warsaw, Poland; 4 Department of Pharmaceutical Biology and Medicinal Plant Biotechnology, Faculty of Pharmacy, Medical University of Warsaw, 1 Banacha St., 02-097 Warsaw, Poland; agnieszka.pietrosiuk@wum.edu.pl (A.P.); katarzyna.syklowska-baranek@wum.edu.pl (K.S.-B.)

**Keywords:** *Boraginaceae* rosmarinic acid, lithospermic B acid, abiotic stresses, chemical profile, in vitro cultures, total phenolic and total flavonoid content

## Abstract

To cope with environmental harmful conditions, plant cells developed adaptive strategy that involves production of a wide variety of complex secondary metabolites. The spectrum and quantity of biosynthesized compounds in specific plant species is determined by its genotype, tissue, developmental and physiological stage and environmental factors. This phenomenon was used to exploit the potential of anatomical and hairy root cultures of *Rindera graeca* to produce bioactive compounds. Cultivated in vitro roots were subjected to abiotic stresses i.e., drought or coldness. Next the extract profiling was performed using HPLC-PDA-ESI-HRMS method, as well quantitative determination of caffeic, rosmarinic and lithospermic B acids, that were present in all root extracts. Phenolic acids, flavonoids and iridoids represent the major groups of compounds detected in chemical profiles growing under various conditions roots. The highest number of phytochemicals was determined in roots subjected to coldness. Lithospermic B acid proved to be the most abundant compound in all investigated extracts. Among applied abiotic stress factors it was demonstrated that coldness affected to the most secondary metabolites production. The results of current study suggest that root cultures of *R. graeca* could serve as a new and abundant source of lithospermic B acid.

## 1. Introduction

In vitro plant cell platforms are continuously explored for application in the biosynthesis of secondary metabolites used as active ingredients of medicines and cosmetics [1,2,3]. The process of production and accumulation of secondary metabolites is affected by many factors, internal e.g., genetic and biochemical as well as external that is environmental which in turn could and influence the plant metabolome [4]. The environmental factors exert a fundamental effect on the biosynthetic capacities of plant cells that could be transferred to in vitro culture conditions and enable the development of efficient biotechnological approaches to enhance the productivity of bioactive compounds in vitro up to cost-effective levels.

*Rindera graeca* (A.DC.) Boiss. & Heldr. (Boraginaceae) is an endemic Greek species growing on rocky slopes at the attitudes of 1500–2300 m [5]. This species is recognized as rare species and placed on the ICUN Red List of Threatened Plants [6]. The chemical profile of aerial parts [7], as well as shoots and roots cultivated in vitro [8,9,10,11] of this species, has been investigated. These studies revealed the presence of phenolic compounds, pyrrolizidine alkaloids, naphthoquinone shikonin-type compounds and among them rinderol, a potent cytotoxic agent [7,11,12]. Rinderol production was optimized in root cultures of *R. graeca* and its proapoptotic activity was demonstrated [13]. Other various biological activities were also reported for plants of *Rindera* genus, including anti-inflammatory [14], anti-viral [15], and antimicrobial attributed to the presence of essential oils distilled from aerial part [16], in addition the latter were also demonstrated for methanolic and hexane extracts of shoots and hairy roots of *R. graeca* cultivated in vitro as well as rinderol [11].

Plants synthetize a large and diverse group of organic compounds known as secondary metabolites or secondary products. These compounds are often found only in some plant species or a related group of species, while the primary metabolites are found throughout the plant kingdom. For many years the importance of most secondary plant metabolites was unknown. These compounds were considered non-functional end products of metabolism [17]. Currently, many secondary metabolites are recognized as having important ecological functions in plants, such as protecting plants from being eaten by herbivores and against infections by pathogens, or as attractants for pollinators and distribute seeds by animals, and as plant-plant competition agents [17,18].

It has been known for many years that the synthesis and accumulation of metabolites is significantly dependent on growth conditions such as temperature, light, water and nutrient availability, etc. The influence of the environment on the secondary metabolism has also been demonstrated, e.g., various stress factors influence the metabolic pathways responsible for the synthesis of secondary metabolites, leading to their accumulation [18]. Most of the studies that have analyzed the content of secondary metabolites are a comparative analysis between stressed and non-stressed plants, covering only one stress factor in a manner. However, in nature, there are various interferences among many stress factors, such as the increase in light intensity is mainly correlated with elevated temperatures and reduced water availability, as well as associated with higher soil salt level. It has been shown in a wide range of experiments that plants exposed to drought stress do indeed accumulate higher concentrations of secondary metabolites. For example, in response to stress, there is an increase in simple and complex phenols, and many terpenes. The content of nitrogen-containing secondary metabolites such as alkaloids, cyanogenic glycosides and glucosinolates is also increased in response to environmental stresses. There is therefore no doubt that the application of drought stress often increases the concentration of some secondary metabolites. However, it should be taken into account that drought stress also restricts the growth of most plants. Therefore, as a simple and obvious explanation of this effect, it is very often given that under drought stress conditions the same amounts of natural products are synthesized and stored in plants as under normal conditions, but—due to the reduction in biomass—their concentration increase [17,18,19].

In the current study, the treated with cold and drought stress factors roots of *R. graeca* cultivated in vitro were subjected to the analysis of their secondary metabolite profile using the HPLC-HR-MS method, as well as quantitative assessment of the most abundant phenolic compounds was performed. Additionally, using atomic absorption spectrometry concentration of main plant macro- and microelements such as Ca, Mg, Na, K, Fe and Mn was measured.

## 2. Materials and Methods

### 2.1. Root Cultures

Three root lines of *Rindera graeca* were subjected for investigation in the current study: an anatomical root line (RgAR), and two hairy root lines (RgTR7 and RgTR17). Root cultures were established by Sykłowska-Baranek et al. [10]. Briefly, the RgAR root line was initiated by cutting off anatomical roots developing on the basis of shoots, hairy root lines were obtained as a result of infection performed with *Agrobacterium rhizogenes* 15,834. All root cultures were performed in a 250 mL Erlenmeyer flask containing 50 mL of liquid hormone-free DCR medium [20] and routinely subcultured every four weeks. The cultures were maintained at 23 ± 1 °C in the dark at 105 rpm on an INFORS gyratory shaker 105 rpm (INFORS AG, Bottmingen, Switzerland).

#### Experimental Design

The 28-day old roots were subjected to drought and cold stress. Each of roots line before stress treatment were transferred to fresh DCR medium.

Each culture of specific root line was divided into three groups: (i) one group was cultivated for 14 days in unchanged conditions (non-treated) and was set as control; (ii) second group was subjected to low temperature of 10 °C cold stress for 14 days; (iii) third group was subjected for 14 days to drought stress which was induced by medium supplementation with 10% of polyethylene glycol (PEG) 6000. PEG was dissolved in DCR medium and then filtered by Nalgene™ Rapid-Flow™ Sterile Single Use Bottle Top Filters. Roots of the control and drought stressed groups were grown at 23 ± 1 °C in the dark at 105 rpm on an INFORS gyratory shaker. For low temperature treatments, roots were transferred to a chamber at 10 °C, in the dark at 105 rpm on an INFORS gyratory shaker.

The elicitation lasted 14 days, next roots were collected, gently pressed on filter paper, and weighted to determine the fresh weight (FW). Afterward, the roots were lyophilized and their dry weight (DW) was recorded. Also the 28-day old roots that was used as a starting material (day “0”) for stress experiments were collected, lyophilized and subjected to comparative phytochemical analysis.

### 2.2. Extraction of Plant Material

The powdered lyophilized roots (100 mg) were extracted using ultrasonic bath with 100% methanol (4 × 5 cm^3^) for 1 h at 25 ± 5 °C. Afterwards the samples were collected and evaporated to dryness under reduced pressure and stored at −20 °C. before analysis. Prior to flavonoids and phenols content measurement the dry residue was dissolved in 80% methanol. Whereas before HPLC-PDA-ESI-HRMS analysis samples were dissolved in 100 % methanol hypergrade for LC-MS LiChrosolv^®^ (Merck; Darmstadt, Germany).

### 2.3. HPLC-PDA-ESI-HRMS Analysis

#### 2.3.1. Chemicals

Methanol HPLC LC-MS Grade (CAS no. 67-56-1) and acetic acid (CAS no. 64-19-7) of HPLC Grade were purchased in Merck (Darmstadt, Germany). Methanol (CAS no. 67-56-1) for plant material extraction purchased in Avantor Performance Materials (Poland). Standard substances of rosmarinic (CAS no. 20283-92-5), chlorogenic (CAS no. 327-97-9), caffeic (CAS no. 331-39-5), ferulic (CAS no. 1135-24-6), 3,5-dicaffeoyl-quinic (CAS no. 2450-53-5), sinapic (CAS no. 530-59-6) and *p*-coumaric (CAS no. 501-98-4) acids were purchased in Sigma-Aldrich (Poznań, Poland); 5-*O*-feruoylouinic acid (CAS no. 1135-24-6) was purchased in LCG Standards Poland. Lithospermic B acid (CAS 115939-25-8), shikonin (CAS no. 517-88-4), acetylshikonin (CAS no. 24502-78-1), isobutyrylshikonin (CAS no. 52438-12-7), deoxyshikonin (CAS no. 43043-74-9) and isovalerylshikonin (CAS no. 52387-14-1) were purchased in ChemFaces (Wuhan, China).

#### 2.3.2. HPLC-PDA-ESI-HRMS Analysis

A Shimadzu Prominence high-performance liquid chromatograph (HPLC) was used coupled with a LCMS-IT-TOF mass spectrometer (Shimadzu Shimadzu Europa GmbH, Duisburg, Germany), equipped with an ion trap (IT), a time-of-flight (TOF) detector and an electrospray ionization (ESI) source. Mass spectra were recorded in the positive and negative ion modes using LCMSsolution software (Shimadzu Shimadzu Europa GmbH, Duisburg, Germany).

Conditions for HPLC separation and detection of extracts were as follows: column Kinetex C_18_, 2.6 µm, 2.1 mm × 100 mm (Phenomenex, Torrance, CA, USA), injection volume: 3 µL, oven column temperature: 40 °C, flow rate: 0.2 mL/min, analysis duration: 75 min, PDA detection at wavelengths λ = 200–800 nm. The mobile phase consisted of (A) water with the addition of 0.2% CHCOOH and (B) methanol. The following gradient was applied: 0–10 min 5% B, 10–30 min 5→50% B, 30–35 min 50→50% B, 35–55 min 50→95% B, 55–60 min 95% B, 60–62 min 95→5% B, equilibrium time—13 min in 5% B.

Conditions for the mass spectrometer were as follows: polarity positive and negative, mass range *m*/*z* 100–1000 Da in both modes, ion accumulation time: 10 ms in MS1 experiments and 25 ms in MS2 experiments, interface temperature: 220 °C, heat block temperature: 220 °C, nebulizing gas flow: 1.5 L/min, drying gas pressure: 100 kPa, IS: +4.5 kV (positive mode) and IS: −3.0 kV (negative mode), collision energy in MS2 experiments: 25–35%.

The TOF detector of the LCMS-IT-TOF mass spectrometer for high resolution mass spectrometry experiments (HRMS) was calibrated with mixture of standard compounds. For all standard samples mass spectra and fragmentation mass spectra were acquired for identification and confirmation of compounds presented in the methanolic extracts. In that case a HRMS experiment was also used for confirmation of molecular formula. In all HRMS experiments a difference between theoretical and measurement *m*/*z* value was below 5 ppm. For the unknown compounds a HRMS experiment was the only one method, which was applied for prediction of the most likely molecular formula.

#### 2.3.3. Standard Sample Preparation

For the calibration curve six calibration standard samples were prepared in the form of a mixture consisting of caffeic acid (CA), rosmarinic acid (RA) and lithospermic B acid (LBA). Concentrations of acids were as follows: caffeic acid in a range of 3.46–111.11 µg/mL, rosmarinic acid in a range of 3.82–122.22 µg/mL, and lithospermic B acid in a range of 4.38–1.26 µg/mL. All acids were prepared by independent dissolving about 1 mg of each acid in 1 mL of methanol and prepared standard mixtures with concentrations around 1 mg/mL were used for calibration mixture preparation.

The methanolic extract was prepared by dissolving in 300 µL of methanol and spinning on vortex, and the supernatant was transferred to an HPLC injection vial.

#### 2.3.4. HPLC Method Validation

The developed method was validated in terms of linearity, specificity, precision, accuracy (recovery) as well as precision and accuracy of Limit of quantification (LOQ).

Analytical specificity was assessed by comparison of UV chromatograms recorded for a blank sample, standard sample and test sample (Figure S1a).

To asses linearity of the assay, six-level calibrators were analyzed. The calibration curve was established by the linear fit of the peak area ratio versus concentration. For each acid an independent calibration curve was established (Figure S1b–d). In the case of caffeic acid a calibration curve crosses zero.

LOQ was determined as the lowest concentration used for calibration curve preparation with accuracy within accuracy (recovery) within ±20% of true value and precision below 5%.

Assay precision and accuracy (recovery) were determined by sevenfold analysis of the test sample and test sample spiked with standards, respectively. In accuracy assays, seven individual test samples with low concentration of endogenous acids were spiked with known amount of caffeic, rosmarinic and lithospermic B acid. Recovery was expressed as a percentage of increased concentration and true added value of acid. The results of method validation are presented in Figure S1.

### 2.4. Determination of Total Flavonoid Content (TFC)

Total flavonoid content was determined colorimetrically based on the reaction following procedures from Pękal and Pyrzyńska [21] with modifications described by Śliwińska et al. [17]. Briefly, extracts or standard (quercetin, QE), were mixed with 5% of sodium nitrate. After 5 min of incubation, 2% aluminum chloride were added and allowed to incubate for another 5 min, after which, 1 M sodium hydroxide were added to the mixture. The evaluation of absorbance for TPC calculations was measured spectrophotometrically at 425 nm. Results are reported as mg of QE equivalents per 1 g of drought weight (mg QE/g DW) using the regression equation determined from the standard curve: y = 0.0021x + 0.0072, r^2^ = 0.9937.

### 2.5. Determination of Total Phenolic Compounds Content (TPC)

The total phenolic compounds content was determined colorimetrically based on the Folin-Ciocalteu [16] with some modification as described Śliwińska et al. [17]. Samples of each extract or standard (gallic acid, GAE) were mixed with the Folin-Ciocalteu reagent, shaked, and mixed with 7% sodium carbonate. All reactions were done in triplicates. A standard GAE curve was prepared as a comparative reference. Results are reported as mg of GAE equivalents per 1 g of drought weight (mg GAE/g DW) using the regression equation determined from the standard curve: y = 0.0088x − 0.0846, r^2^ = 0.9947. The evaluation of absorbance for TPC calculations was measured spectrophotometrically at 765 nm.

### 2.6. Determination of Ions Concentration

The following ions concentrations: Ca, Mg, Na, K, Fe and Mn, were determined in roots by atomic absorption spectrometry (SpektrAA 300, Varian, Mulgrave, Australia) following wet digestion of 50 mg of oven dried plant tissue samples in 5 mL of 69% HNO_3_ at 140 °C.

### 2.7. Statistical Analysis

Nine biological replicates per treatment and three for time zero cultures were used for growth, TPC and TFC statistical analysis. Whereas for analytical examination six replicates per treatment and three for time zero cultures were used. Determination of ion concentration was performed based on five replicates. Data represents mean values ± standard deviation (SD). The statistical significance between means was assessed using the Kruskal-Wallis one-way analysis of variance performed with STATISTICA 13.1 PL (StatSoft, Kraków, Poland) software. Significance between groups was further estimated using the Mann-Whitney U test. A probability of *p* < 0.05 was considered significant. Pair-wise metabolite-antioxidant effects correlations were calculated by Pearson’s correlation coefficient test.

## 3. Results and Discussion

### 3.1. Biomass, Total Phenols and Flavonoids as Well as Ions Concentration

The effect of abiotic stress on biomass accumulation varied according to root line. Only in RgAR roots both stresses caused growth of fresh weight (FW) by 56% and 5% in response to drought or cold stress, respectively. In opposite, under cold stress condition the decrease in FW by 55% and 42 % was observed in RgTR7 and RgTR17 root lines, respectively (Table 1).

Dry weight analysis indicates that only in response to cold stress biomass decreased by 39% (RgAR), 51% (RgTR7) and 39% (RgTR17) in comparison to control cultures (Table 1).

In current study the inhibition of root biomass accumulation under cold stress was observed. The decreasing biomass in response to this stress was also reported e.g., in rice [22]. Further, the lack of changes in biomass growth under drought stress could be probably linked with the adaptation effect of this plant species to its natural environment, which is rocky mountains of Greece. Usually, chilling and freezing stresses limits the growth and development of plants, and reduce primary metabolism and cause e.g., a violation of the stability of proteins or protein complexes and a decrease in enzymatic activity [23].

Generally in the plants, the same amounts of ions are absorbed or metabolites are synthesized and accumulated, as under well-watered conditions, but—due to the reduction in biomass—their concentration simply is elevated [17]. Both used stressors did not significantly influence on the changes in the ions concentration between stressed roots and respective controls (Table 2).

**Figure 1:**
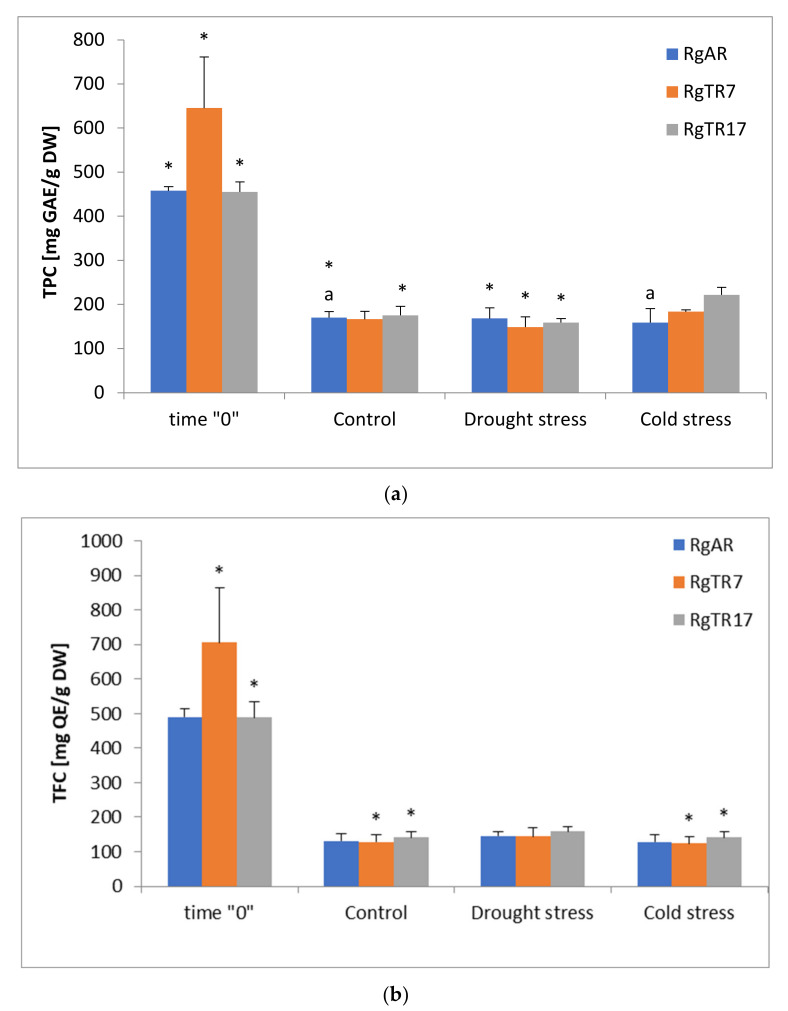
(**a**) Total phenolic (TPC) and (**b**) total flavonoid (TFC) content determined in *R. graeca* roots cultivated under various conditions. RgAR—anatomical roots; RgTR7—hairy root line TR7; RgTR17—hairy root line TR17; time “0”—28-day-old roots at time of inoculation; Control—roots cultivated without any treatment for 14 days; Drought stress—roots treated by drought stress for 14 days; Cold stress—roots treated by cold stress for 14 days. The same letters indicate statistically significant differences (*p* ≤ 0.05) in relation to control within the same root lines between treatments. Asterisks (*) indicate statistically significant differences (*p* ≤ 0.05) in relation to time “0” within the same root lines between treatments.

Total phenolic compounds concentration (TPC) in unstressed RgAR and RgTR17 were significantly lower by about 65% than in RgAR “0” and RgTR17 “0”, respectively (Figure 1a). In response to drought stress TPC significantly decrease by 65–77% in each of examined root lines in compare to RgAR “0”, RgTR7 “0” and RgTR17 “0”. The lowest significant changes in phenols concentration in the RgAR root line was observed after cold stress treatment and was lower by 6% than in RgAR “0” and RgAR.

In the anatomical root line both stresses did not cause changes in total flavonoids concentration. The concentration of this compounds significantly decreased in RgTR7 and drought stressed RgTR7 by about 72% in compare to RgTR7 “0”. Similarly in RgTR7 and drought stressed RgTR7 were observed decrease of total flavonoids by 51–62% in compare to RgTR7 “0” (Figure 1b).

The results of TPC, TFC and HPLC analysis are consistent. Three selected for quantitative determination phenolic acids are part of the total pool of phenolic compounds that is estimated to exceed 8000 molecules [24], among others are phenolic acids and flavonoids. The highest concentration of investigated compounds determined in roots used for inoculation could be attributed to observed in plant in vitro cultures distinct lag phase when biosynthesis of secondary metabolites is performed at very low levels [25], which was also observed under conditions of present study. The abiotic stress factors applied acted for 14 days and did not affected significantly TPC and TFC accumulation in relation to control.

### 3.2. HPLC-PDA-ESI-HRMS Analysis

The HPLC-PDA-ESI-HRMS analysis of methanolic extracts derived from roots cultivated in control and drought or cold stress treated root cultures was performed to determine 16 standard compounds (Table 3). The major constituents of investigated extracts were caffeic (CA), rosmarinic (RA) and lithospermic B acid (LAB) and their concentration in root extracts was determined. The validation parameters elaborated for quantitative HPLC analysis of three phenolic acids, i.e., CA, RA and LAB are presented in Figure S1.

For all standard samples mass spectra and fragmentation mass spectra were acquired for identification and confirmation of compounds presented in the methanolic extracts. In addition, HRMS experiment was also used for confirmation of molecular formula. Further, the profiling of extracts by HRMS method was carried out, and prediction of the most likely molecular formula of detected compounds was done (Table S1–S3). In all HRMS experiments a difference between theoretical and measurement *m*/*z* value was below 5 ppm (Table S4). The compounds annotation was performed based on the Pub Chem database. The structures were proposed in accordance with recorded HRMS measurements that consisted of finding the most suitable molecular formulas with mass accuracy below 5 ppm. Moreover, in the Pub Chem database, a lot of additional information like provenance and compounds class were published, which were also used for the identification of compounds found in methanolic extracts. All of the proposed structures were known in literature and were also detected in various parts of different plants.

Irrespectively root line, the highest content of investigated phenolic acids was determined in 28-day old roots that is at time zero (Table 4). The quantitative analysis of CA, RA and LAB in root extracts revealed that LBA was the most abundant phenolic acid accumulated. At this time point the LBA content was the highest in RgTR7 roots (106.07 ± 10.65 mg/g DW) and was almost 1.7- and over 1.2-fold higher than in RgAR and RgTR17 roots, respectively. RA concentration was also the highest in roots of RgTR7 line, although its concentration was lower than LAB content almost 5-, 3- and 8-fold in RgAR, RgTR7 and RgTR17 roots, respectively. CA was present in the lowest concentration in investigated root extracts.

In all examined root lines stresses caused increase in concentration of CA. In response to drought or cold, in the RgAR concentration of this compound grew by 100% and 167%, respectively. In the RgTR7 both stresses caused growth of CA by 67%. The level of C A in RgTR17 increased by 125% and 100% after drought and cold stress treatment, respectively. In compare to CA, RA production presented different dynamic of changes. Only in cold stressed roots concentration of RA increased by 163% (RgAR) and 152% (RgTR17) in compare to respective control. The concentration of this acid in cold stressed RgTR7 decrease by 33% than in control.

In response to cold stress the high growth of LAB by 237% (RgTR7) and 268% (RgTR17) than in controls was observed whereas level of this compounds in RgAR increased 15-fold than in unstressed RgAR (Table 4). The production of LBA was mostly reported for plants of Lamiaceae family [25,26], with one study describing its and RA accumulation in hairy root cultures of *Lithospermum erythrorhizon* [27]. Nevertheless, the yield of LBA reported in current study substantially exceeds its previously reported productivity.

Abiotic stresses, including drought and low temperature ones, are broadly used to improve production of secondary metabolites or induce de novo their biosynthesis [28]. The significant role in production of secondary metabolites under stress condition is attributed to generation of oxidative stress defense response [29,30]. In turn to cope with excessive production of reactive oxygen species (ROS) generated induction of secondary metabolites biosynthesis is initiated.

**Figure 2:**
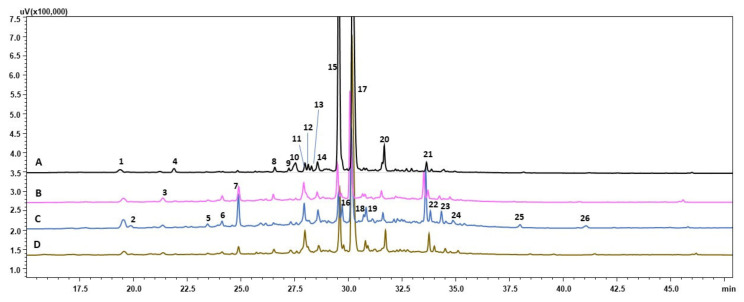
HPLC-PDA chromatograms (wavelength 320 nm) of RgAR line root extracts:(A) 28-day-old (time zero); (B) 14-day old untreated roots-control; (C) roots treated 14 days with drought stress; (D) roots treated 14 days with cold stress.

**Figure 3:**
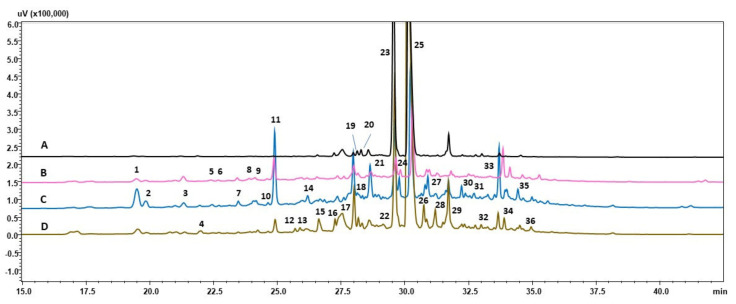
HPLC-PDA chromatograms (wavelength 320 nm) of RgTR7 line root extracts: (A) 28-day-old (time zero); (B) 14-day old untreated roots-control; (C) roots treated 14 days with drought stress; (D) roots treated 14 days with cold stress.

**Figure 4:**
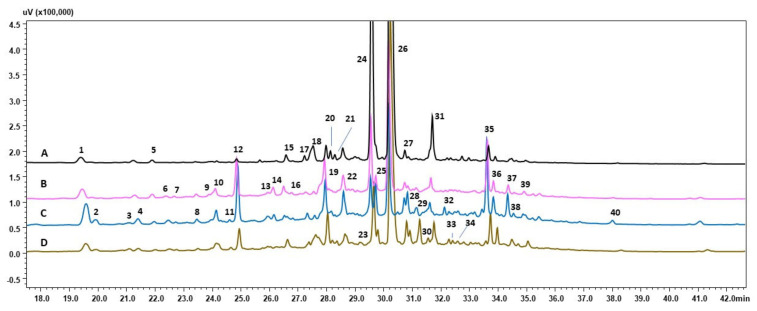
HPLC-PDA chromatograms (wavelength 320 nm) of RgTR17 line root extracts: (A) 28-day-old (time zero); (B) 14-day old untreated roots-control; (C) roots treated 14 days with drought stress; (D) roots treated 14 days with cold stress.

The present study analysis of metabolome showed variation in chemical profiles between investigated root lines treated with various stress conditions (Figure 2, Figure 3 and Figure 4; Table 5, Table 6 and Table 7). However, the majority of metabolites biosynthesized by root of the same line were similar and the patter of compounds detected was similar. The main groups of secondary metabolites produced in response to stresses applied belongs to flavonoids, phenolic compounds and iridoids, that is metabolites that were reported to play crucial role in plant cell protection against detrimental environmental factors [28,31,32]. Many of compounds detected in chemical profiles of investigated root lines, as well quantitative analysis of CA, RA and LBA are reported for the first time in *R. graeca* root extracts [7,11]. The results of present study are not consistent with the previous report describing chemical prolife of *R. graeca* roots cultivated in vitro [11], that noted lack of LAB and chlorogenic acid. While in present study LAB was the major secondary metabolite accumulated in roots. However, RA yield determined in roots cultivated under conditions of present study was considerably lower than that quantified in *R. lanata* aerial parts [15]. Further, in present study no quercetin 3-rutinoside-7-rhamnoside or pyrrolizidine alkaloids were detected which were also previously found in *R. graeca* [7,11] and other species of this genus [33,34]. None of the compounds detected in investigated root extracts obtained under conditions of this study was documented before in *Rindera* genus. In examined *R. graeca* root extracts no shikonin derivatives were detected (Table 3). This phenomena was earlier reported in cell suspension cultures of *L. erythrorhizon* [35]. Authors suggest that in specific culture conditions, in LBA and shikonin biosynthetic pathway, in which they share its early steps, the phenylpropanoid unit is further favorably used for LBA synthesis. Nevertheless under conditions of this study in two samples of 28-day old roots: RgAR and hairy of RgTR17 line was detected rinderol, a furano-naphthoquinone compound demonstrating antiapoptotic potential [13]. Previously rinderol was determined both in RgTR7 and RgTR17 28-old-day root lines cultivated in vitro [11], but in further subcultures its biosynthesis was induced only in cultures carried out on polyurethane rafts [11]. Such gradual loss of biosynthetic capacity, could be attributed to genetic and epigenetic variation during long-term cultivation in vitro [36,37], which is believed to be the main cause of decrease in secondary metabolites production abilities.

The results of current study suggest that root cultures of *R. graeca* could serve as a new and abundant source of LBA, the phenolic acid exhibiting various biological activities like lowering blood pressure [38], cytoprotective effects on pancreatic β-cells [39,40] and cardioprotective properties [41]. HPLC-PDA-ESI-HRMS analysis revealed differences in chemical profiles of investigated root lines that could be connected with their genetic diversity as well as be connected with stress factors used. Among abiotic stressors the cold had the most impact on accumulation of three selected phenolic acids, however effect of both used abiotic factors on their biosynthesis was not considerable. In conclusion, *R. graeca* roots, hairy and anatomical, are an interesting plant material for further phytochemical and biological exploitation. Further investigations are needed to identified other detected in root extracts molecules.

## Figures and Tables

**Table 1:** Biomass [mg] of *R. graeca* roots cultivated under various conditions.

Treatment	28 Day Old Roots-Time “0”			Control			Drought Stress			Cold Stress		
	Root Line											
FW/DW	RgAR	RgTR7	RgTR17	RgAR	RgTR7	RgTR17	RgAR	RgTR7	RgTR17	RgAR	RgTR7	RgTR17
FW	1835.7 ± 103.0 *	1040.3 ± 387.6 *	2068.1 ± 239.7 *	2138.7 ± 573.8 ^a^*	3550.7 ± 854.7 ^b^*	4708.4 ± 421.1 ^c^*	3344.3 ± 454.3 *	3280.8 ± 879.9 *	3732.6 ± 525.8	2241.2 ± 254.7 ^a^	1683.6 ± 685.2 ^b^	2740.0 ± 408.5 ^c^
DW	360.9 ± 23.6	229.9 ± 92.1	359.5 ± 13.5	664.3 ± 79.6	621.7 ± 144.2	767.8 ± 35.7	654.9 ± 61.4	646.2 ± 153.1	700.3 ± 62.2	408.1 ± 41.7	305.7 ± 134.7	470.2 ± 55.5

FW—fresh weight; DW—dry weight; RgAR—anatomical roots; RgTR7—hairy root line TR7; RgTR17—hairy root line TR17; time “0”—28-day-old roots at time of inoculation; Control—roots cultivated without any treatment for 14 days; Drought stress—roots treated by drought stress for 14 days; Cold stress—roots treated by cold stress for 14 days. Means denoted with the same letter or asterisk are statistically significant (*p* < 0.05). The same letters indicate statistically significant differences (*p* ≤ 0.05) in relation to control within the same root lines between treatments. Asterisks (*) indicate statistically significant differences (*p* ≤ 0.05) in relation to time “0” within the same root lines between treatments.

**Table 2:** Ions concentration [ppm/g DW] in *R. graeca* roots cultivated under various conditions.

Treatment	28 Day Old Roots-Time “0”			Control			Drought Stress			Cold Stress		
	Root Line											
Ion	RgAR	RgTR7	RgTR17	RgAR	RgTR7	RgTR17	RgAR	RgTR7	RgTR17	RgAR	RgTR7	RgTR17
Ca	3.37 ± 0.81	1.64 ± 0.12	2.81 ± 0.55	2.64 ± 0.42	2.12 ± 0.20	2.90 ± 0.17 ^a^	2.50 ± 0.25	2.27 ± 0.34	2.24 ± 0.40	2.50 ± 0.31	1.90 ± 0.19 ^a^	2.66 ± 0.27
Mg	1.36 ± 0.27	0.76 ± 0.03	1.29 ± 0.29	1.17 ± 0.19	1.06 ± 0.14	1.41 ± 0.09 *^a,b^	1.04 ± 0.11	0.87 ± 0.15 ^a^	0.90 ± 0.17	1.06 ± 0.06	0.87 ± 0.06 ^b^	1.20 ± 0.11
Na	2.53 ± 0.16	2.51 ± 0.28	2.37 ± 0.07	2.47 ± 0.11	2.33 ± 0.18	2.19 ± 0.07	2.20 ± 0.10	2.11 ± 0.70	2.09 ± 0.07 ^a,b^	2.82 ± 0.18 ^a^	2.78 ± 0.36 ^b^	2.52 ± 0.12
K	11.29 ± 1.09	11.25 ± 0.29	11.11 ± 1.85	13.37 ± 0.68 ^a,b^	12.35 ± 0.53	11.42 ± 0.54	10.93 ± 0.41	10.29 ± 0.77 ^a^	9.57 ± 1.15 ^b^	10.91 ± 2.57	12.89 ± 0.70	12.74 ± 1.41
Fe	0.35 ± 0.04	0.30 ± 0.04	0.27 ± 0.01	0.26 ± 0.02	0.21 ± 0.02	0.23 ± 0.02	0.22 ± 0.03	0.23 ± 0.04	0.17 ± 0.02 ^a,b,c^	0.29 ± 0.05 ^a^	0.33 ± 0.05 ^b^	0.29 ± 0.03 ^c,b^
Mn	0.38 ± 0.04	0.33 ± 0.01	0.34 ± 0.02	0.31 ± 0.03	0.29 ± 0.01	0.30 ± 0.01	0.27 ± 0.01	0.25 ± 0.03 ^a,b^	0.22 ± 0.03	0.34 ± 0.01 ^a^	0.32 ± 0.02	0.34 ± 0.02 ^b^

RgAR—anatomical roots; RgTR7—hairy root line TR7; RgTR17—hairy root line TR17; time “0”—28-day-old roots at time of inoculation; Control—roots cultivated without any treatment for 14 days; Drought stress—roots treated by drought stress for 14 days; Cold stress—roots treated by cold stress for 14 days. Means denoted with the same letter or asterisk are statistically significant (*p* < 0.05). Asterisks (*) indicate statistically significant differences (*p* ≤ 0.05) in relation to control within the same root lines between treatments. The same letters indicate statistically significant differences (*p* ≤ 0.05) between different root lines.

**Table 3:** The presence of standard compounds in *Rindera graeca* root extracts determined by HPLC-PDA-ESI-HRMS analysis.

Treatment	28 Day Old Roots-Time “0”			Control			Drought Stress			Cold Stress		
	Root Line											
Compound	RgAR	RgTR7	RgTR17	RgAR	RgTR7	RgTR17	RgAR	RgTR7	RgTR17	RgAR	RgTR7	RgTR17
Caffeic acid	+	+	+	+	+	+	+	+	+	+	+	+
Chlorogenic acid	−	−	−	−	−	−	−	−	−	−	−	−
*p*-coumaric acid	−	−	−	−	−	−	−	−	−	−	−	−
5-*O*-feruoylo-quinic acid	−	−	−	−	−	−	−	−	−	−	−	−
Sinapic acid	−	−	−	−	−	−	−	−	−	−	−	−
3,5-dicaffeoyl-quinic acid	−	−	−	−	−	−	−	−	−	−	−	−
Rosmarinic acid	+	+	+	+	+	+	+	+	+	+	+	+
Lithospermic acid	−	−	+	−	−	+	−	−	+	−	−	+
Lithospermic B acid	+	+	+	+	+	+	+	+	+	+	+	+
Shikonin	−	−	−	−	−	−	−	−	−	−	−	−
Acetylshikonin	−	−	−	−	−	−	−	−	−	−	−	−
Isobutyrylshikonin	−	−	−	−	−	−	−	−	−	−	−	−
Deoxyshikonin	−	−	−	−	−	−	−	−	−	−	−	−
Isovalerylshikonin	−	−	−	−	−	−	−	−	−	−	−	−
Dimethylacrylshikonin	−	−	−	−	−	−	−	−	−	−	−	−
Rinderol	+	−	+	−	−	−	−	−	−	−	−	−

RgAR—anatomical roots; RgTR7—hairy root line TR7; RgTR17—hairy root line TR17; time “0”—28-day-old roots at time of inoculation; Control—roots cultivated without any treatment for 14 days; Drought stress—roots treated by drought stress for 14 days; Cold stress—roots treated by cold stress for 14 days.

**Table 4:** Phenolic acid content [mg/g DW] in *R. graeca* roots cultivated under various conditions.

Treatment	28 Day Old Roots-Time “0”			Control			Drought Stress			Cold Stress		
	Root Line											
Compound	RgAR	RgTR7	RgTR17	RgAR	RgTR7	RgTR17	RgAR	RgTR7	RgTR17	RgAR	RgTR7	RgTR17
Caffeic acid	0.20 ± 0.01	0.01 ± 0.01	0.24 ± 0.04	0.03 ± 0.005	0.03 ± 0.005 *	0.04 ± 0.01 *	0.06 ± 0.15	0.05 ± 0.01 *	0.09 ± 0.02 *	0.08 ± 0.02	0.05 ± 0.01	0.08 ± 0.01 ^a^
Rosmarinic acid	12.74 ± 0.12	33.69 ± 15.11	10.97 ± 1.24	0.95 ± 0.08	0.90 ± 2.45	0.52 ± 0.23	0.87 ± 0.10	0.99 ± 0.60	0.48 ± 0.24	2.50 ± 0.98 *	0.60 ± 0.14^*^	1.31 ± 0.50
Lithospermic B acid	63.17 ± 17.68	106.07 ± 10.65	87.77 ± 14.71	2.05 ± 0.43 ^a^	1.68 ± 0.79 ^b^	2.01 ± 0.32 ^c^	2.13 ± 0.34	1.76 ± 0.67	1.71 ± 0.08 ^c^	31.78 ± 7.08 ^a,^*	5.67 ± 2.37 ^b,^*	7.39 ± 1.12 ^c,^*

RgAR—anatomical roots; RgTR7—hairy root line TR7; RgTR17—hairy root line TR17; time “0”—28-day-old roots at time of inoculation; Control—roots cultivated without any treatment for 14 days; Drought stress—roots treated by drought stress for 14 days; Cold stress—roots treated by cold stress for 14 days. The same letters indicate statistically significant differences (*p* ≤ 0.05) in relation to control in specific phenolic acid content within the same root line. Asterisks (*) indicate statistically significant differences (*p* ≤ 0.05) in specific phenolic acid content among root lines within the same treatment.

**Table 5:** HPLC-PDA-ESI-HRMS data on detected compounds in RgAR root extracts derived from various culture conditions.

Peak No.	Tr	[M–H]^-^	Molecular Formula	Compound	Conditions
1	19.39	179	C9H8O4	Caffeic acid	DZ; control; DS; CS
2	19.88	329 341	C15H22O8 C15H18O9	Bartsioside Caffeic acid 3-glucoside	DS
3	21.22	431	C20H24N4O7	Unidentified	DZ; control; DS; CS
4	21.90	375	C18H16O9	Limocitrol	DZ
5	23.46	499	C22H28O13	Haploperoside	DS
6	24.13	509 553	C33H18O6 C27H22O13	Unidentified Unidentified	control; DS
7	24.89	269 313 627 715	C16H14O4 C17H14O6 C34H28O12 C36H28O16	Imperatorin Crisimaritin Unidentified Dehydrorabdosiin	DZ; control; DS; CS
8	26.58	733	C54H22O4	Unidentified	DZ; control; DS; CS
9	27.24	515	C26H32N2O9	Strictosidinic acid?	DZ; DS; CS
10	27.54	436	C25H31N3O4	N1,N10-Bis(p-coumaroyl)spermidine	DZ
11	27.99	537 545	C27H22O12 C32H34O8	Globoidnan B Vittarin E	DZ; control; DS; CS
12	28.14	439 (2-)	C28H32N16O8	Unidentified	DZ
13	28.29	435 521	C20H20O11 C24H26O13	Irisxanthone Iridin	DZ
14	28.57	359 369 483	C18H16O8 C21H18N6O or C20H22N2O5 C22H28O12	Irigenin Unidentified or Apabetalone Rubinaphthin B/7-methyl-1,4,5-naphthalenetriol-4-[xylosyl-(1→6)-glucoside]/MEGxp0_002017	DZ; control; DS; CS
15	29.55	359	C18H16O8	Rosmarinic acid	DZ; control; DS; CS
16	29.71	447 461 627	C22H24O10 C22H22O11 C28H36O16	Sakuranin or Androechin Azalein or Tectoridin Piloside A	DS; CS
17	30.11	717	C36H30O16	Lithospermic B acid	DZ; control; DS; CS
18	30.71	383	C21H24N2O5 or C22H20N6O	Unidentified	DZ; control; DS; CS
19	30.83	335 461 557	C17H20O7 C22H22O11 C25H34O14	Unidentified Azalein Peujaponiside or Macrophylloside D	DZ; control; DS; CS
20	31.66	551	C28H24O12	Schizotenuin F	DZ; control; DS; CS
21	33.63	465 613	C22H26O11 C29H42O14	Curculigoside Unidentified	DZ; control; DS; CS
22	33.82	611 669 765	C29H40O14 C35H24O14 C48H46O9	Unidentified S-(+)-skyrin-6-O-alpha- arabinofuranoside Unidentified	control; DS; CS
23	34.33	449	C22H26O10	Auriculoside or 4-methoxyphlorizin	DS; CS
24	34.87	451	C23H32O9	Unidentified	DS; CS
25	37.99	303	C16H16O6	Unidentified	DS
26	41.07	215	C13H12O3	Unidentified	DS

DZ—28-old day roots (day zero); Control—14-day-old untreated roots; DS—roots treated with drought stress for 14 days; CS—roots treated with cold stress for 14 days.

**Table 6:** HPLC-PDA-ESI-HRMS data on detected compounds in RgTR7 root extracts derived from various culture conditions.

Peak No.	Tr	[M–H]^-^	Molecular Formula	Compound	Sample No.
1	19.43	179	C9H8O4	Caffeic acid	DZ, control, DS, CS
2	19.78	329 341	C15H22O8 C15H18O9	Bartsioside Caffeic acid 3-glucoside	control, DS, CS
3	21.27	431	C19H28O11	Zizybeoside I	control, DS, CS
4	21.90	519	C29H28O9	Unidentified	DS, CS
5	22.40	271 297 299 415 553	C15H12O5 C16H10O6 C14H12N4O4 C18H24O11 C25H30O14	Naringenin Irilone or Trifoliol or 3,8-dihydroxy-1-methylanthraquinone-2-caroxylic acid Unidentified Regaloside L or Carbomethoxyferuoyl sorbitol Isoligusrosidic acid or Aquilarisinin	control
6	22.69	431 483 579	C18H24O12 C22H28O12 C32H36O10	Griselinoside Rubinaphthin B Unidentified	control, DS
7	23.41	373 399 475 519	C16H22O10 C18H24O10 C23H24O11 C24H24O13	Unidentified Regaloside Cirsimarin Eujambolin or Purifolin	control, DS, CS
8	23.90	499	C22H28O13	Haploperoside	control, DS
9	24.23	337 467 509 553	C16H18O8 C15H32O16 C26H22O11 C27H22O13	Coumaroylquinic acid I or II Unidentified Unidentified Unidentified	control, DS, CS
10	24.57	501 597 699	C24H22O12 C26H30O16 C33H32O17	Malonyldaidzin Swertiapuniside Unidentified	control, DS, CS
11	24.83	269 313 627 671 715	C16H14O4 C17H14O6 C34H28O12 C30H28N2O16 C36H28O16	Imperatorin Crisimaritin Unidentified Unidentified Dehydrorabdosiin	control, DS, CS
12	25.70	531	C38H28O3	Unidentified	CS
13	25.89	501	C22H30O13	Ferulic acid rutinoside	CS
14	26.13	547 581 729	C25H28N2O12 C26H30O15 C36H26O17	Unidentified Gentiabavaroside or Sophodibenzenoside A Unidentified	DS
15	26.63	733	C36H30O17	Unidentified	CS
16	27.26	515 581 717	C26H32N2O9 C26H30O15 C36H30O16	Strictosidinic acid? Gentiabavaroside or Sophodibenzenoside A Rabdosiin	DZ, control, CS
17	27.54	436 479	C25H31N3O4 C22H24O12	N1,N10-Bis(p-coumaroyl)spermidine	DZ, control, DS, CS
18	28.01	537	C27H22O12	Globoidnan B	DZ, control, DS, CS
19	28.17	459 879	C20H28O12 C38H36ON6O19	Paeonolide or Apiopaeonoside Unidentified	DZ, control, DS, CS
20	28.32	435	C20H20O11	Irisxanthone or Homomangiferin or Swertianolin	DZ, CS
21	28.57	369 521 715	C19H16O9 C24H26O13 C36H28O16 or C42H24O11	Unidentified Rosmarinic acid hexoside Dehydrorabdosiin	DZ, control, DS, CS
22	29.14	435	C20H20O11	Irisxanthone or Homomangiferin or Swertianolin	CS
23	29.65	359 493	C18H16O8 C26H22O10	Rosmarinic acid Salvianolic acid A	DZ, control, DS, CS
24	29.71	447 627	C22H24O10 C28H36O16	Sakuranin or Androechin Piloside A	control, DS
25	30.30	717	C36H30O16	Lithospermic B acid	DZ, control, DS, CS
26	30.74	383	C21H24N2O5	Unidentified	control, DS, CS
27	30.83	461	C22H22O11	Azalein	control, DS, CS
28	31.20	445	C22H22O10	Swertisin or Glycitin or Sissotrin	DZ, control, DS, CS
29	31.71	551	C28H24O12	Schizoteniun F	DZ, control, DS, CS
30	32.15	493 641	C26H22O10 C31H30O15	Dihydrogloboidnan A Unidentified	DS, CS
31	32.64	311	C16H8O7	Unidentified	control, DS, CS
32	32.99	635	C38H36O9	Unidentified	DS, CS
33	33.66	613	C29H42O14	Unidentified	control, DS, CS
34	33.90	451 765	C23H32O9 C41H50O14	Unidentified Unidentified	control, DS, CS
35	34.36	449	C22H26O10	Auriculoside	control, DS, CS
36	34.95	275 313 451	C15H16O5 C17H14O6 C23H32O9	Unidentified Pityrogrammin Unidentified	control, DS, CS

DZ—28-old day roots (day zero); Control—14-day-old untreated roots; DS—roots treated with drought stress for 14 days; CS—roots treated with cold stress for 14 days.

**Table 7:** HPLC-PDA-ESI-HRMS data on detected compounds in RgTR17 root extracts derived from various culture conditions.

Peak No.	Tr	[M–H]^-^	Molecular Formula	Compound	Sample No.
1	19.58	179	C9H8O4	Caffeic acid	DZ, control, DS, CS
2	19.91	329 341	C15H22O8 C15H18O9	Bartsioside Caffeic acid 3-glucoside	control, DS, CS
3	21.10	431	C19H28O11	Zizybeoside I	DZ, control, DS, CS
4	21.40	499	C22H28O13	4-Methylumbelliferyl-beta-D-lactoside or Haploperoside	DZ, control, DS,CS
5	21.97	519	C22H32O14	Segetoside A	DZ, control, DS, CS
6	22.46	271 553	C15H12O5 C25H30O14	Naringenin Isoligusrosidicacid or Aquilarisinin	control, DS, CS
7	22.72	483	C22H28O12	Rubinaphthin B	control, DS, DC
8	23.46	443	C26H20O7	Artomunoxanthentrione	DZ, control, DS, DC
9	23.96	499	C22H28O13	4-Methylumbelliferyl-beta-D-lactoside or Haploperoside	control, DS, DC
10	24.10	509 553	C26H22O11 C27H22O13	Pseudonocardone C Unidentified	control, DS, DC
11	24.58	505	C21H30O14	Echisoside	DZ, control, DS, DC
12	24.85	269 313 627 715	C16H14O4 C17H14O6 C34H28O12 C36H28O16	Unidentified Crisimaritin Unidentified Dehydrorabdosiin	DZ, control, DS, DC
13	25.95	501	C22H30O13	Ferulic acid rutinoside	control, DS
14	26.13	227 547 581 729	C12H12N4O C30H28O10 C26H30O15 C36H26O17	Unidentified 3,5-dihydroxyrottlerin Gentiabavaroside or Sophodibenzenoside A Unidentified	control, DS
15	26.50	733	C36H30O17	Unidentified	DZ, control, DS, DC
16	26.77	439 459 501 534	C22H32O9 C20H28O12 C22H30O13 C21H17NO7	Unidentified Paeonolide or Apiopaeonoside Micromelumoside B Unidentified	control, DS
17	27.23	515	C31H32O7	Pannokin A	DZ, control, DS, DC
18	27.53	436	C25H31N3O4	Unidentified	DZ, control, DS, DC
19	27.93	339 459 537	C18H12O7 C20H28O12 C27H22O12	Grevilline B Paeonolide or Apiopaeonoside Globoidnan B	DZ, control, DS, DC
20	28.14	439(2+)	C60H32O8	Unidentified	DZ, DS, DC
21	28.30	435	C20H20O11	Irisxanthone or Homomangiferin or Swertianolin	DZ, DS, DC
22	28.58	359 483 715	C18H16O8 C22H28O12 C36H28O16	Irigenin Rubinaphthin B or 3,4-dihydrocatalposide Unidentified	DZ, control, DS, DC
23	29.16	435	C20H20O11	Irisxanthone or Homomangiferin or Swertianolin	DZ, DC
24	29.55	359 493 537	C18H16O8 C26H22O10 C27H22O12	Rosmarinic acid Salvianolic acid A Lithospermic acid	DZ, control, DS, DC
25	29.72	419 447 449 627	C20H20O10 C22H24O10 C17H26N2O12 C28H36O16	Isogentisin 3-O-glucoside Sakuranin or Androechin Unidentified Piloside A	control, DS, DC
26	30.17	717	C36H30O16	Lithospermic B acid	DZ, control, DS, DC
27	30.74	383	C26H24O3	Unidentified	DZ, control, DS, DC
28	30.86	335 465 497 533 557 611	C17H20O7 C22H26O11 C16H34O17 C29H26O10 C25H34O14 C27H32O16	Unidentified Curculigoside Unidentified Unidentified Peujaponiside Hydroxysafflor yellow A	DZ, control, DS, DC
29	31.26	445	C22H22O10	Swertisin or Glycitin or Sissotrin	control, DS, DC
30	31.55	475 701	C23H24O11 C35H30N2O14	Crisimarin or Kakkalidone Unidentified	DC
31	31.69	551	C28H24O12	Schizoteniun F	DZ, control, DS, DC
32	32.28	320 335 507 539 641	C31H30O15 C27H44O19 C24H28O12 C25H32O13 C31H30O15	Unidentified Unidentified Specioside or 10-O-cis-p-Coumaorylcatalpol Oleuropein Unidentified	DS, DC
33	32.41	465	C22H26O11	Curculigoside	DC
34	32.59	453	C22H30O19	Unidentified	DS, DC
35	33.67	613	C29H42O14	Unidentified	DZ, control, DS, DC
36	33.90	435 451 669 765	C17H12N10O5 C19H28N6O7 C36H26O14 C41H50O14	Unidentified Unidentified Unidentified Unidentified	DZ, control, DS, DC
37	34.36	449	C22H26O10	Auriculoside	DZ, control, DS, DC
38	34.57	463	C22H24O11	Lanceolin or Scuteamoenoside	DZ, control, DS, DC
39	34.91	451	C23H32O9	Unidentified	DZ, control, DS, DC
40	38.01	303 479 543	C16H16O6 C23H28O11 C29H36O10	3‘-O-Methylcatechin Unidentified Unidentified	DZ, control, DS, DC

DZ—28-old day roots (day zero); Control—14-day-old untreated roots; DS—roots treated with drought stress for 14 days; CS—roots treated with cold stress for 14 days.

## Data Availability

The data presented in this study are available on request from the corresponding author. The data are not publicly available because they are a part of one of co-authors habilitation work and after finishing this work the data will be accessible.

## Supplementary Materials

The following supporting information can be downloaded at: https://www.mdpi.com/article/10.3390/cells11060931/s1, Figure S1: Validation parameters of HPLC method., Table S1: Mass spectra of compounds detected in extracts of RgAR root line; Table S2: Mass spectra of compounds detected in extracts of RgTR7 root line; Table S3: Mass spectra of compounds detected in extracts of RgTR17 root line; Table S4 HRMS data for investigated methanolic extracts.
