# Supplementary material for: HPLC-PDA-ESI-HRMS-Based Profiling of Secondary Metabolites of Rindera graeca Anatomical and Hairy Roots Treated with Drought and Cold Stress
Source: Cells. 2022 Mar 8;11(6):931. doi: 10.3390/cells11060931 (PMC8946546; doi:10.3390/cells11060931)
Supplement: Supplementary file 1 [file cells-11-00931-s001.zip › Figure S1.pdf]

(a)

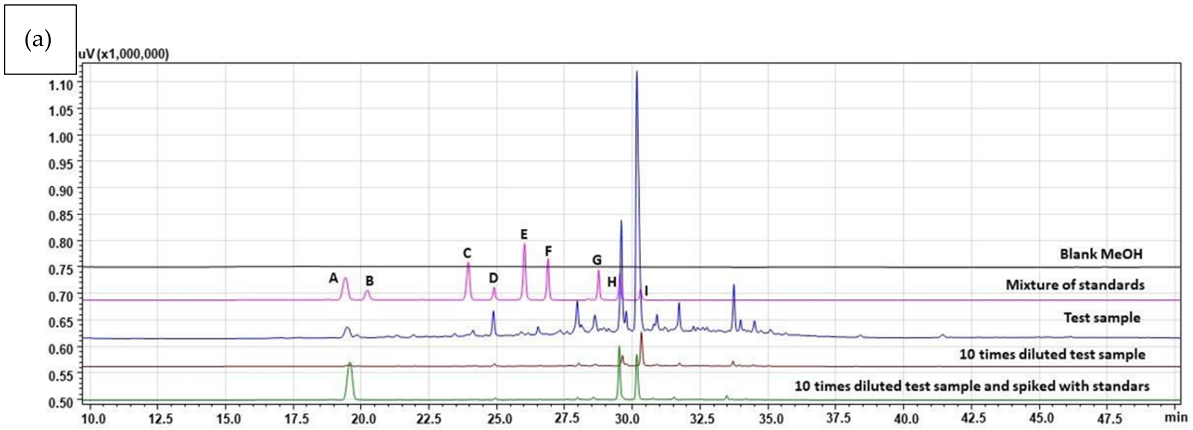

(b)

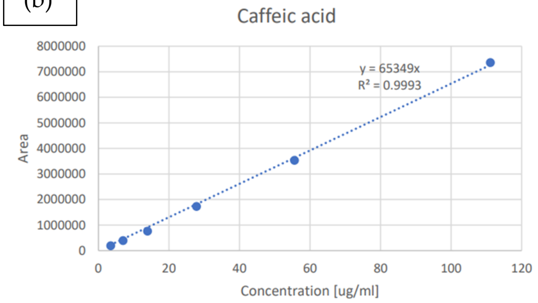

(c)

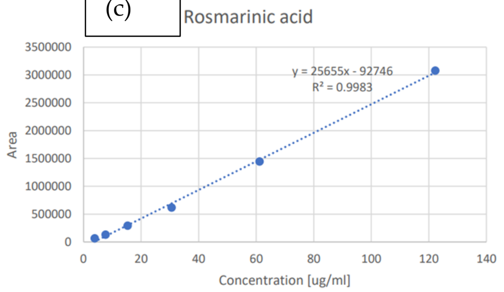

(d)

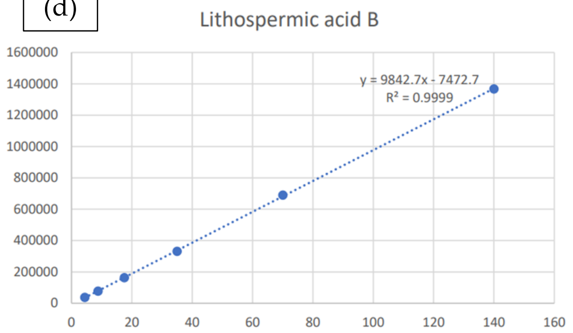

(e)

Standard compound	Regression equations	Correlation coefficient	LOQ (RSD %)	Precision (RSD %)	Recovery [%]
Caffeic acid	y=65349x	0.9993	1.97	1.85	102
Rosmarinic acid	y=25655x-92746	0.9983	2.01	3.77	105
Lithospermic B acid	y=9842.7x-7472.7	0.9999	2.25	3.77	96

Figure S1. (**a**) Assessment of analytical specificity by comparison of UV chromatograms recorded for a blank sample, standard sample and test sample. (**b**) Caffeic acid calibration curve established by the linear fit of the peak area ratio versus concentration; (**c**) Rosmarinic acid calibration curve established by the linear fit of the peak area ratio versus concentration; (**d**) Lithospermic B acid calibration curve established by the linear fit of the peak area ratio versus concentration. (**e**) The results of validation parameters of quantitative HPLC analysis.
