# Supplementary material for: HPLC-PDA-ESI-HRMS-Based Profiling of Secondary Metabolites of Rindera graeca Anatomical and Hairy Roots Treated with Drought and Cold Stress
Source: Cells. 2022 Mar 8;11(6):931. doi: 10.3390/cells11060931 (PMC8946546; doi:10.3390/cells11060931)

HPLC-PDA-ESI-HRMS-Based Profiling of Secondary Metabolites of *Rindera graeca* Anatomical and Hairy Roots Treated With Drought And Cold Stress

Marcin R. Naliwajski [1#], Beata Wileńska[2,3*#], Aleksandra Misicka[2,3], Agnieszka Pietrosik[4] and Katarzyna Sykłowska-Baranek[4]

[1] Department of Plant Physiology and Biochemistry, Faculty of Biology and Environmental Protection, University of Lodz, 12/16 Banacha St., 90-237 Lodz, Poland; marcin.naliwajski@biol.uni.lodz.pl (M.R.N.)

[2] Faculty of Chemistry, University of Warsaw, 1 Pasteura St., 02-093 Warsaw, Poland; bwilenska@chem.uw.edu.pl (B.W.); misicka@chem.uw.edu.pl (A.M.)

[3] Biological and Chemical Research Centre, 101 Żwirki i Wigury St., 02-097 Warsaw, Poland

[4] Department of Pharmaceutical Biology and Medicinal Plant Biotechnology, Faculty of Pharmacy, Medical University of Warsaw, 1 Banacha St, 02-097 Warsaw, Poland; katarzyna.syklowska-baranek@wum.edu.pl (K.S.B.); agnieszka.pietrosiuk@wum.edu.pl (A.P.)

[#] These authors contributed equally to the work.

* Correspondence: bwilenska@chem.uw.edu.pl

Table S1. Mass spectra of compounds detected in extracts of RgAR root line.

Peak number	Mass spectra
Peak 1	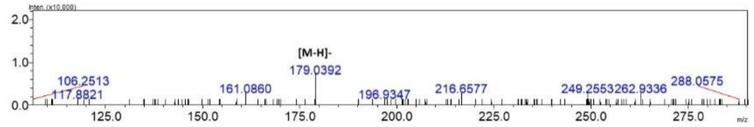
Peak 2	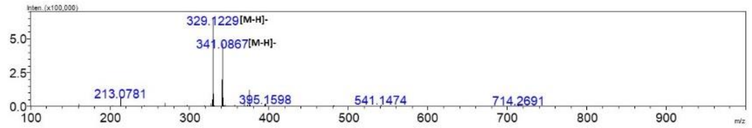
Peak 3	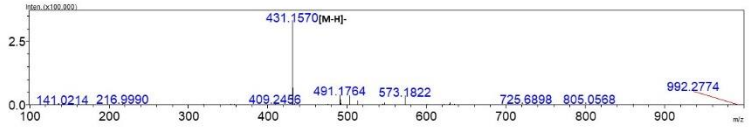
Peak 4	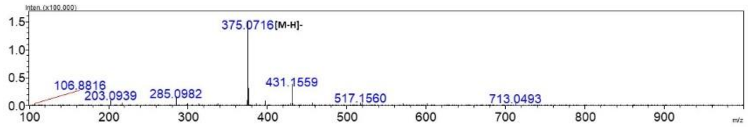
Peak 5	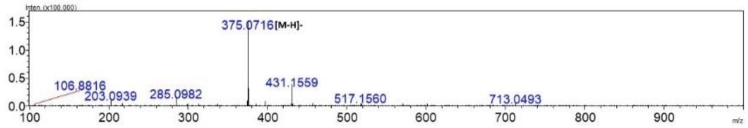
Peak 6	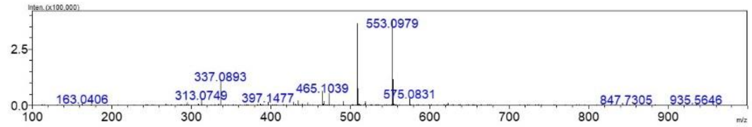 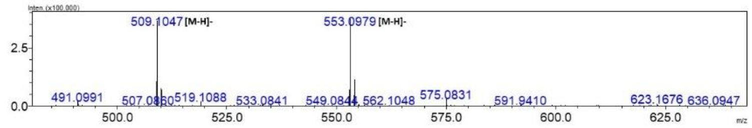
Peak 7	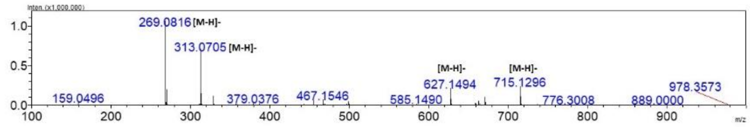
Peak 8	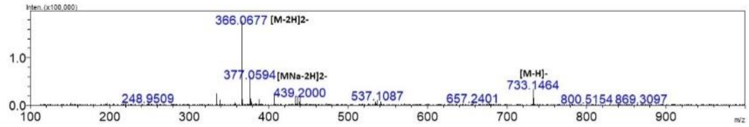
Peak 9	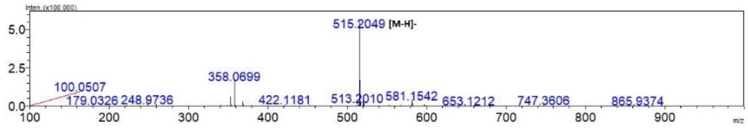
Peak 10	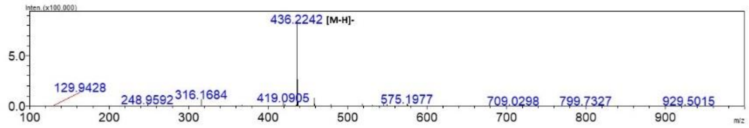

Peak 11

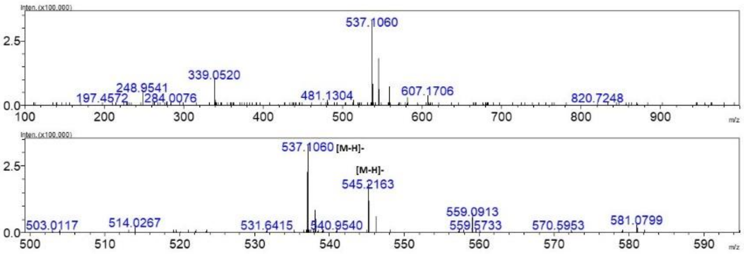

Peak 12

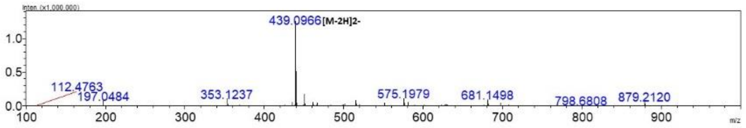

Peak 13

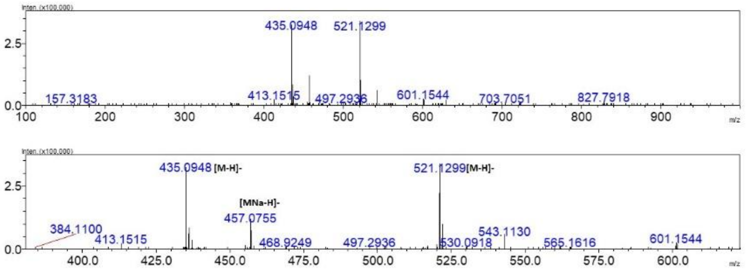

Peak 14

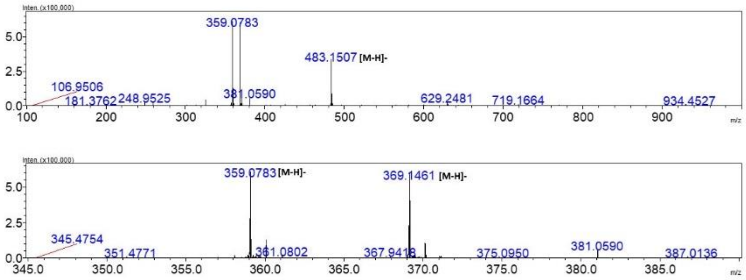

Peak 15

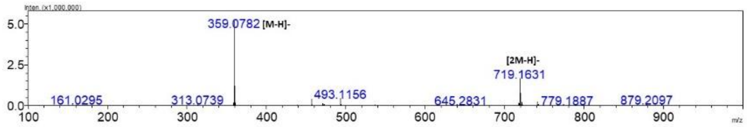

Peak 16

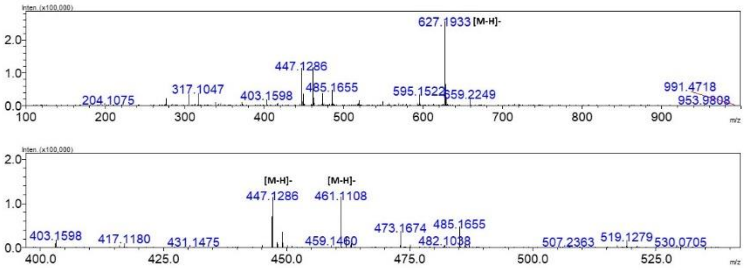

Peak 17

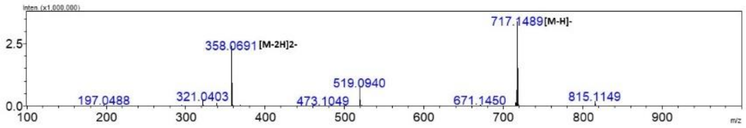

Peak 18

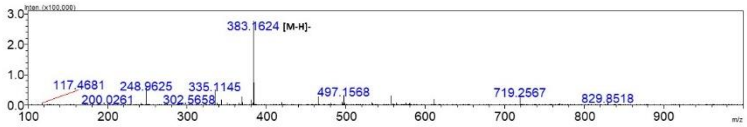

Peak 19

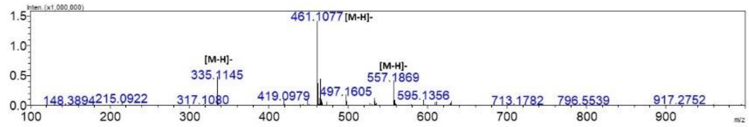

Peak 20

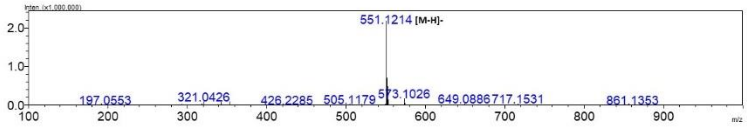

Peak 21

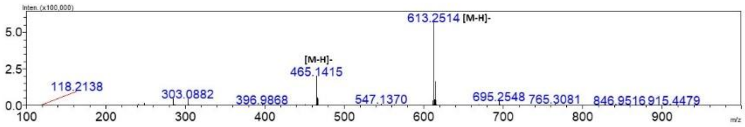

Peak 22

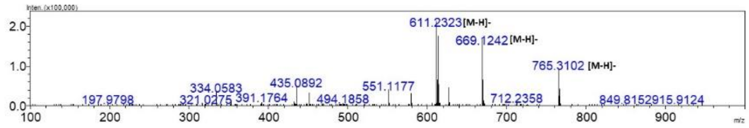

Peak 23

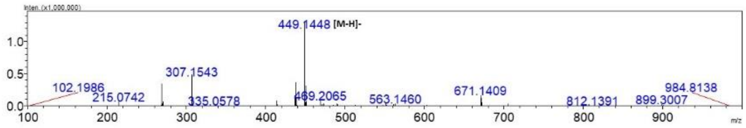

Peak 24

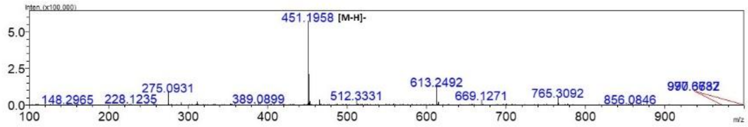

Peak 25

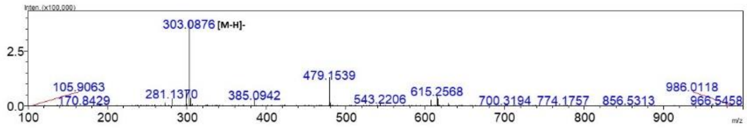

Peak 26

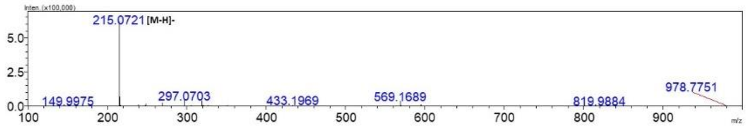

Supplement: Supplementary file 1 [file cells-11-00931-s001.zip › Table S1.pdf]
