# Supplementary material for: HPLC-PDA-ESI-HRMS-Based Profiling of Secondary Metabolites of Rindera graeca Anatomical and Hairy Roots Treated with Drought and Cold Stress
Source: Cells. 2022 Mar 8;11(6):931. doi: 10.3390/cells11060931 (PMC8946546; doi:10.3390/cells11060931)
Supplement: Supplementary file 1 [file cells-11-00931-s001.zip › Table S2.pdf]

# HPLC-PDA-ESI-HRMS-Based Profiling of Secondary Metabolites of *Rindera graeca* Anatomical and Hairy Roots Treated With Drought And Cold Stress

**Marcin R. Naliwajski [1#], Beata Wileńska[2,3*#], Aleksandra Misicka[2,3], Agnieszka Pietrosik[4] and Katarzyna Sykłowska-Baranek[4]**

[1] Department of Plant Physiology and Biochemistry, Faculty of Biology and Environmental Protection, University of Lodz, 12/16 Banacha St., 90-237 Lodz, Poland; marcin.naliwajski@biol.uni.lodz.pl (M.R.N.)

[2] Faculty of Chemistry, University of Warsaw, 1 Pasteura St., 02-093 Warsaw, Poland; bwilenska@chem.uw.edu.pl (B.W.); misicka@chem.uw.edu.pl (A.M.)

[3] Biological and Chemical Research Centre, 101 Żwirki i Wigury St., 02-097 Warsaw, Poland

[4] Department of Pharmaceutical Biology and Medicinal Plant Biotechnology, Faculty of Pharmacy, Medical University of Warsaw, 1 Banacha St, 02-097 Warsaw, Poland; katarzyna.syklowska-baranek@wum.edu.pl (K.S.B.); agnieszka.pietrosiuk@wum.edu.pl (A.P.)

[#] These authors contributed equally to the work.

* Correspondence: bwilenska@chem.uw.edu.pl

**Table S2.** Mass spectra of compounds detected in extracts of RgTR7 root line.

| Peak number | Mass spectra |
| --- | --- |
| Peak 1 | 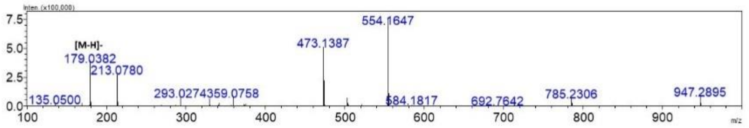 |
| Peak 2 | 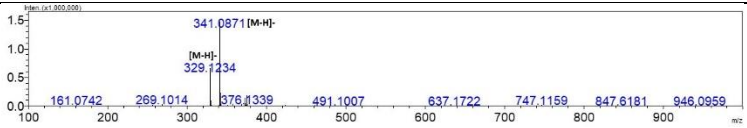 |
| Peak 3 | 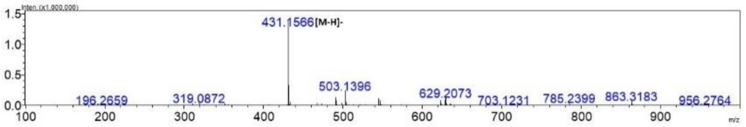 |
| Peak 4 | 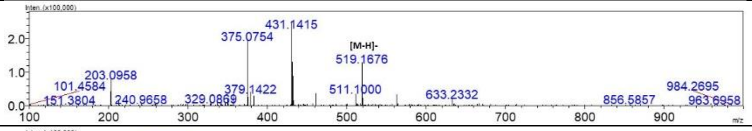 |
| Peak 5 | 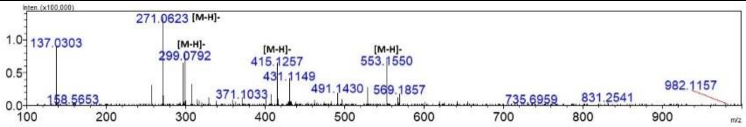 |
| Peak 6 | 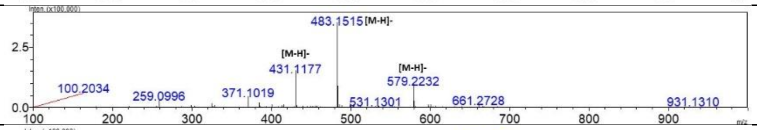 |
| Peak 7 | 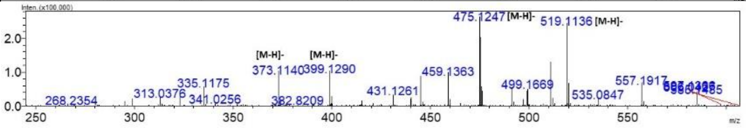 |
| Peak 8 | 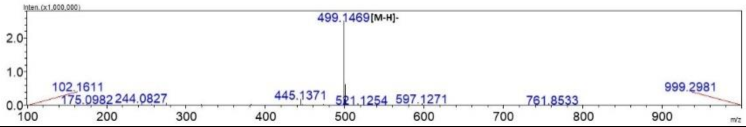 |
| Peak 9 | 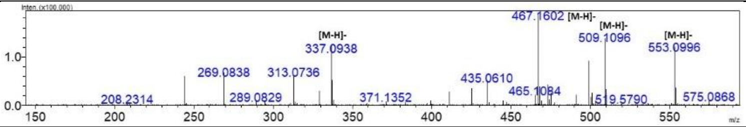 |
| Peak 10 | 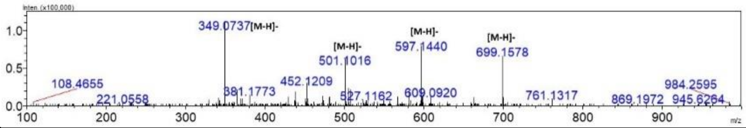 |
| Peak 11 | 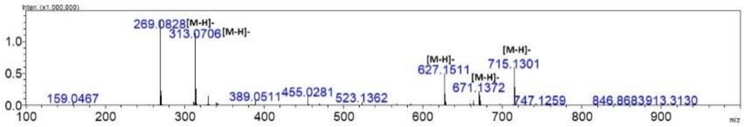 |
| Peak 12 | 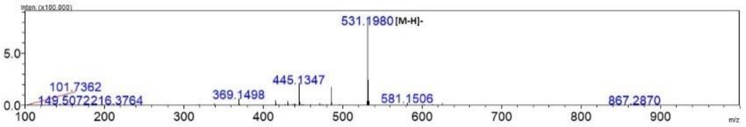 |
| Peak 13 | 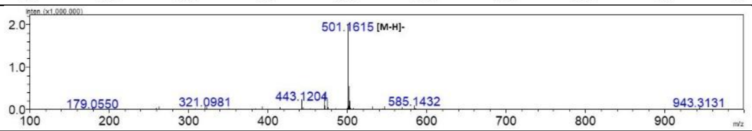 |

Peak 14

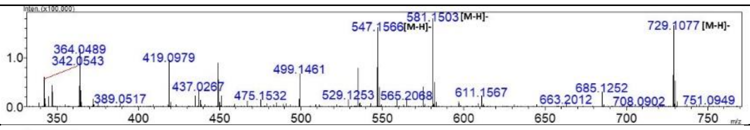


Peak 15

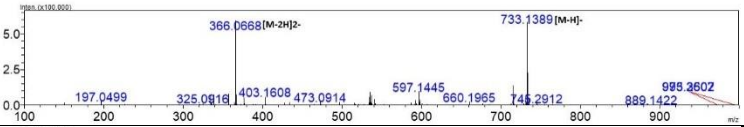


Peak 16

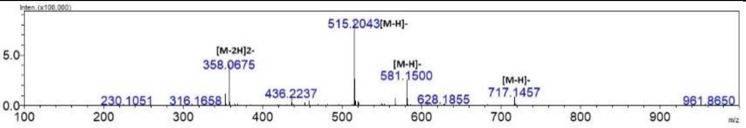


Peak 17

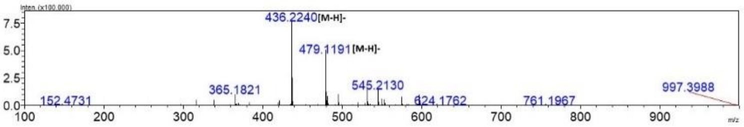


Peak 18

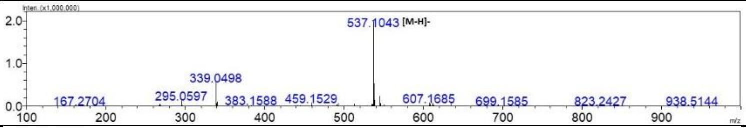


Peak 19

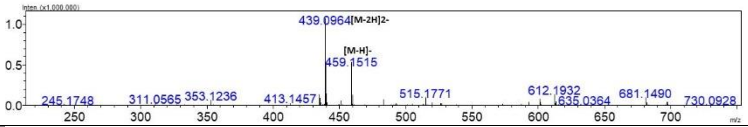


Peak 20

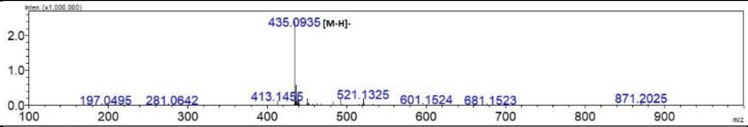


Peak 21

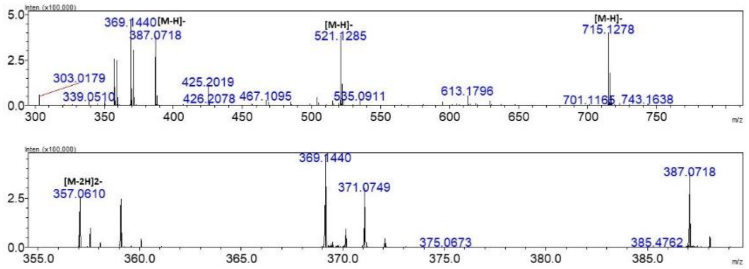


Peak 22

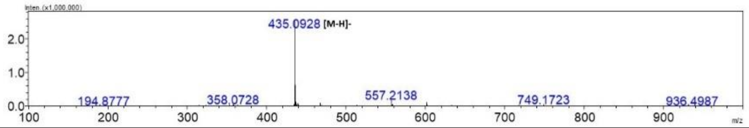


Peak 23

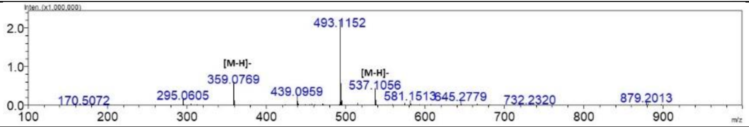


Peak 24

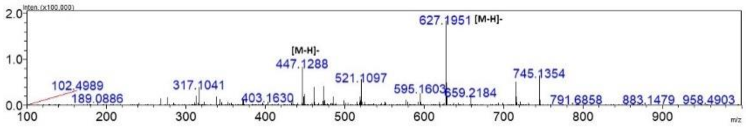


Peak 25

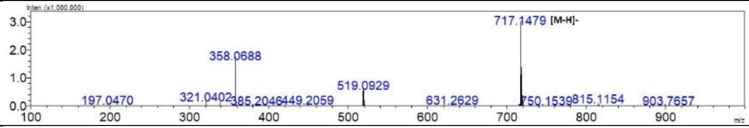


Peak 26

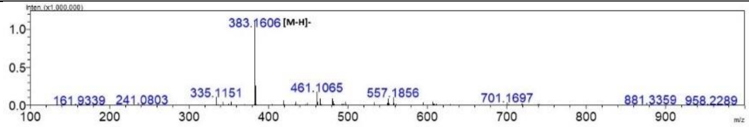

| | |
|---|---|
| Peak 27 | 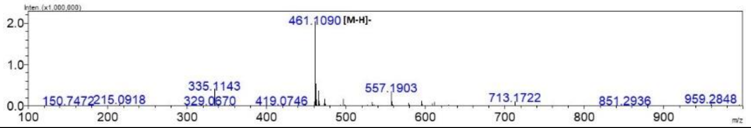 |
| Peak 28 | 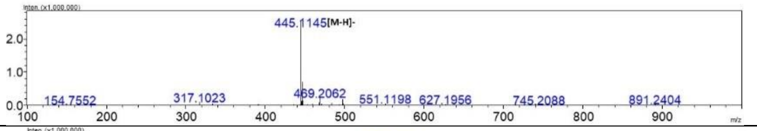 |
| Peak 29 | 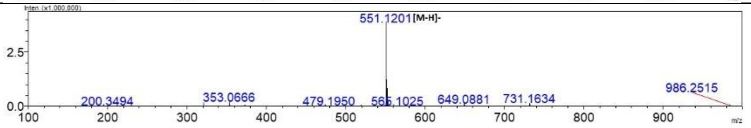 |
| Peak 30 | 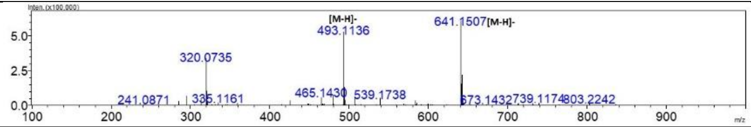 |
| Peak 31 | 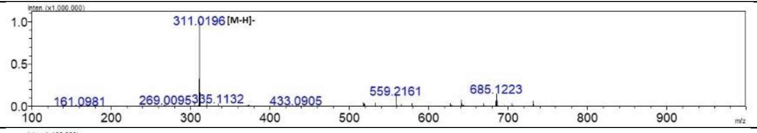 |
| Peak 32 | 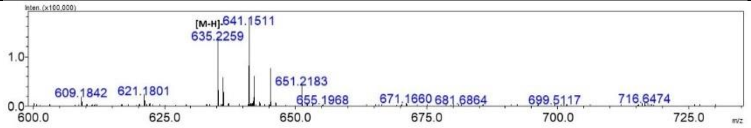 |
| Peak 33 | 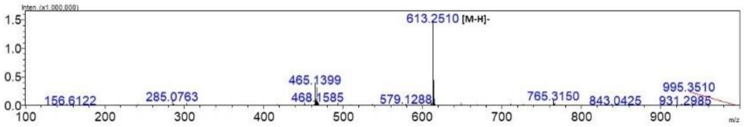 |
| Peak 34 | 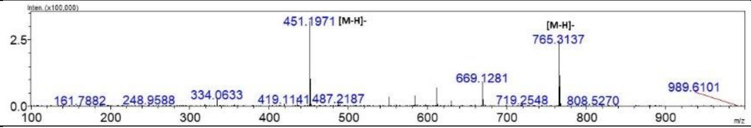 |
| Peak 35 | 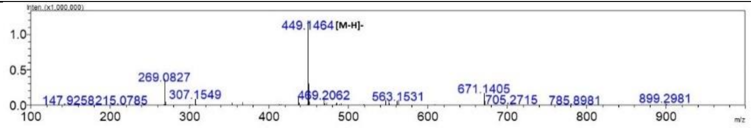 |
| Peak 36 | 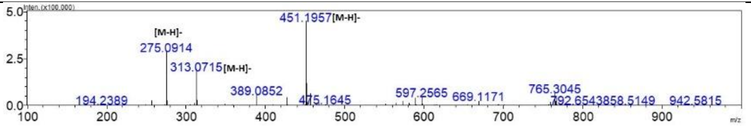 |
