# Supplementary material for: HPLC-PDA-ESI-HRMS-Based Profiling of Secondary Metabolites of Rindera graeca Anatomical and Hairy Roots Treated with Drought and Cold Stress
Source: Cells. 2022 Mar 8;11(6):931. doi: 10.3390/cells11060931 (PMC8946546; doi:10.3390/cells11060931)

# HPLC-PDA-ESI-HRMS-Based Profiling of Secondary Metabolites of *Rindera graeca* Anatomical and Hairy Roots Treated With Drought And Cold Stress

**Marcin R. Naliwajski [1#], Beata Wileńska[2,3*#], Aleksandra Misicka[2,3], Agnieszka Pietrosik[4] and Katarzyna Sykłowska-Baranek[4]**

[1] Department of Plant Physiology and Biochemistry, Faculty of Biology and Environmental Protection, University of Lodz, 12/16 Banacha St., 90-237 Lodz, Poland; marcin.naliwajski@biol.uni.lodz.pl (M.R.N.)

[2] Faculty of Chemistry, University of Warsaw, 1 Pasteura St., 02-093 Warsaw, Poland; bwilenska@chem.uw.edu.pl (B.W.); misicka@chem.uw.edu.pl (A.M.)

[3] Biological and Chemical Research Centre, 101 Żwirki i Wigury St., 02-097 Warsaw, Poland

[4] Department of Pharmaceutical Biology and Medicinal Plant Biotechnology, Faculty of Pharmacy, Medical University of Warsaw, 1 Banacha St, 02-097 Warsaw, Poland; katarzyna.syklowska-baranek@wum.edu.pl (K.S.B.); agnieszka.pietrosiuk@wum.edu.pl (A.P.)

[#] These authors contributed equally to the work.

* Correspondence: bwilenska@chem.uw.edu.pl

**Table S3.** Mass spectra of compounds detected in extracts of RgTR17 root line.

| Peak number | Mass spectra |
|---|---|

Peak 1

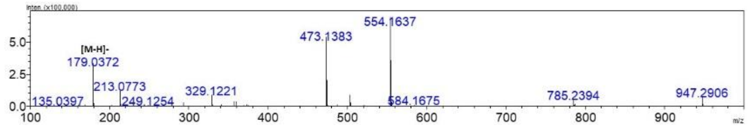


Peak 2

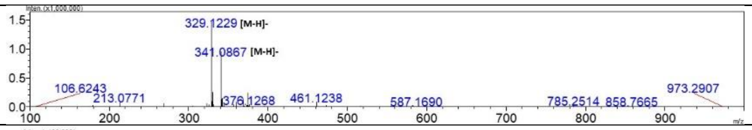


Peak 3

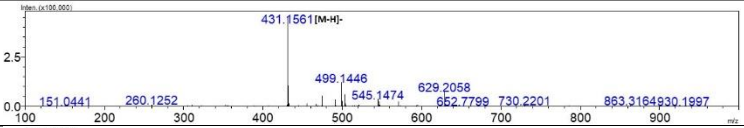


Peak 4

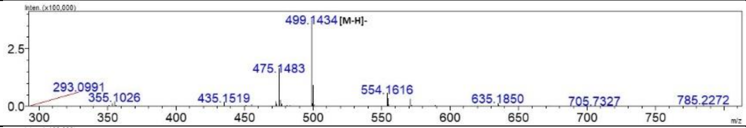


Peak 5

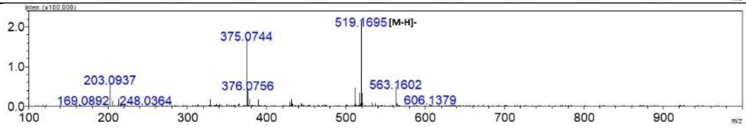


Peak 6

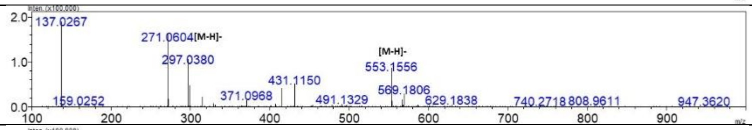


Peak 7

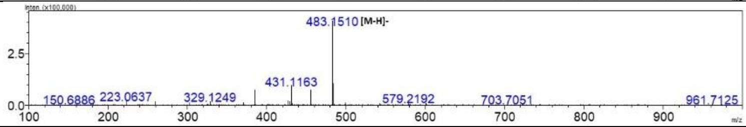


Peak 8

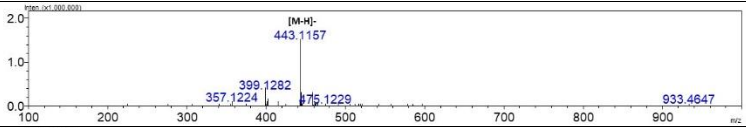


Peak 9

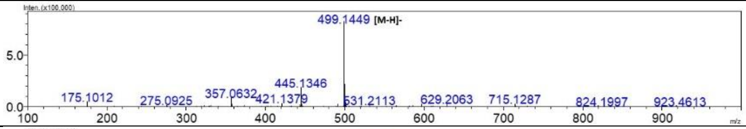


Peak 10

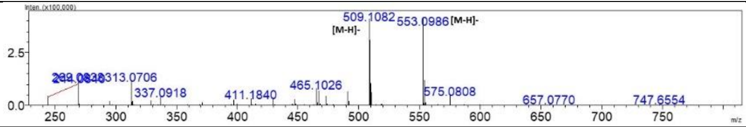


Peak 11

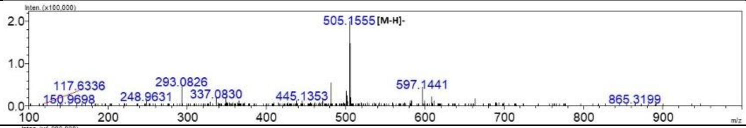


Peak 12

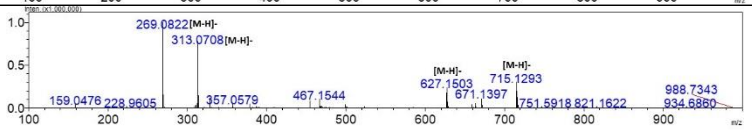


Peak 13

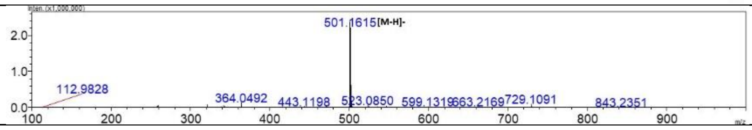

Peak 14

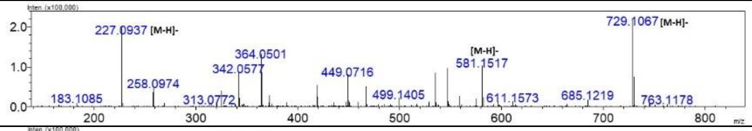


Peak 15

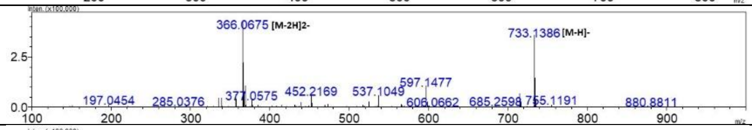


Peak 16

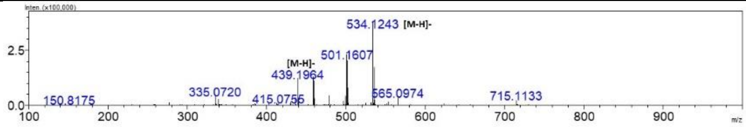


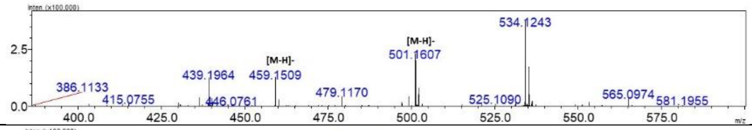


Peak 17

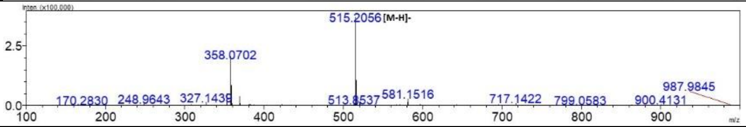


Peak 18

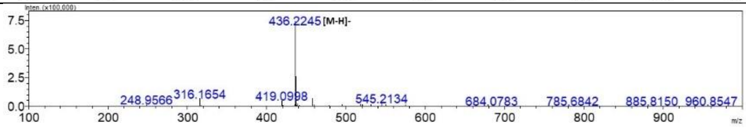


Peak 19

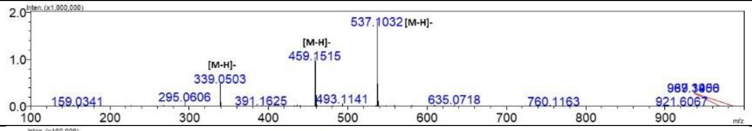


Peak 20

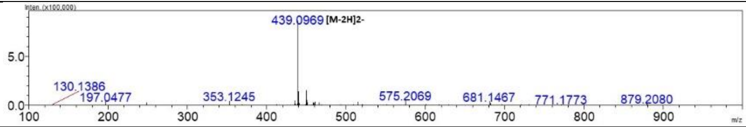


Peak 21

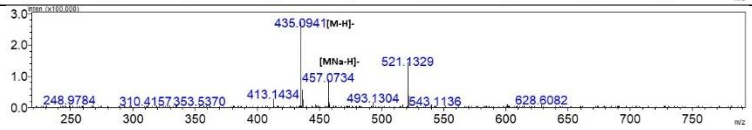


Peak 22

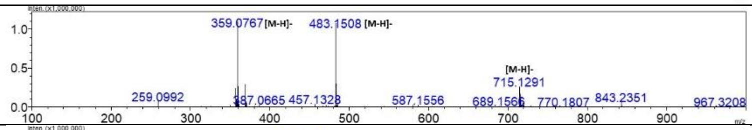


Peak 23

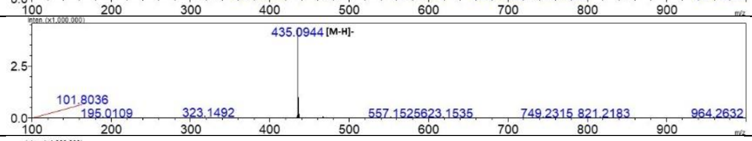


Peak 24

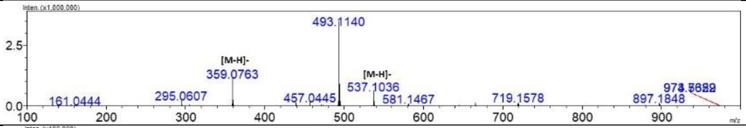


Peak 25

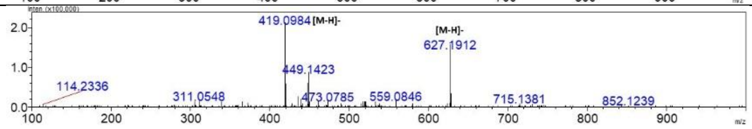


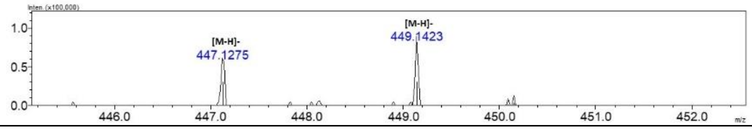

Peak 26

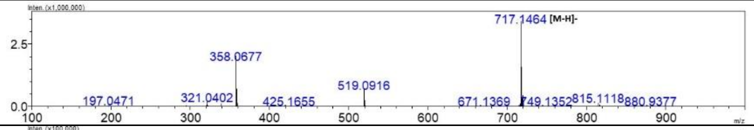


Peak 27

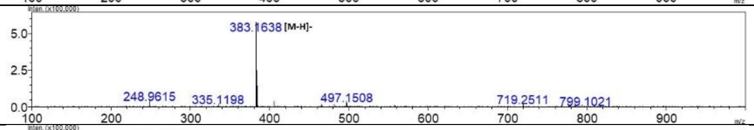


Peak 28

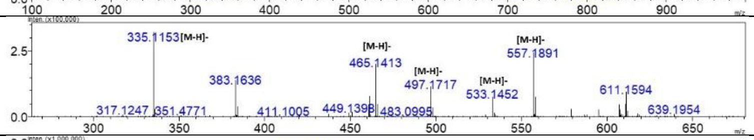


Peak 29

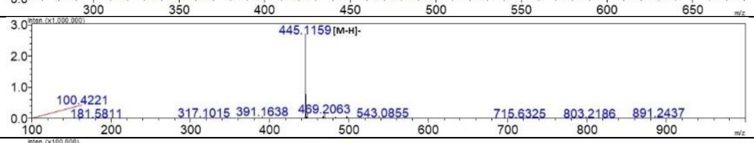


Peak 30

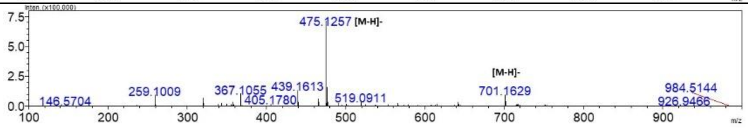


Peak 31

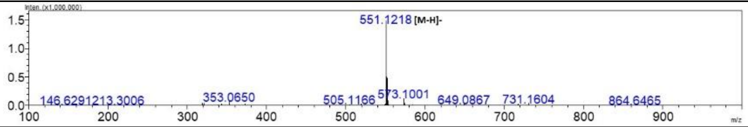


Peak 32

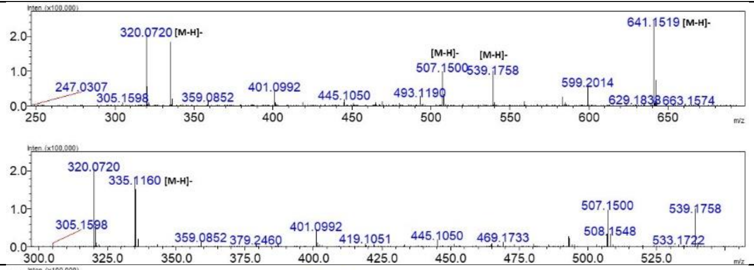


Peak 33

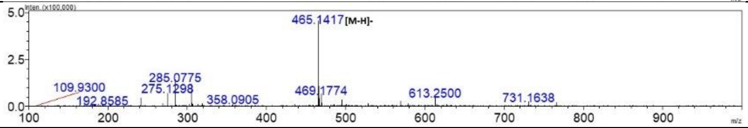


Peak 34

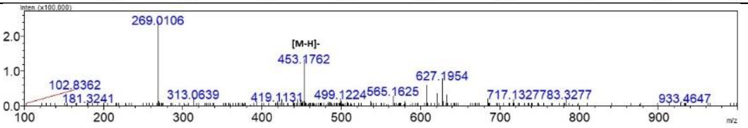


Peak 35

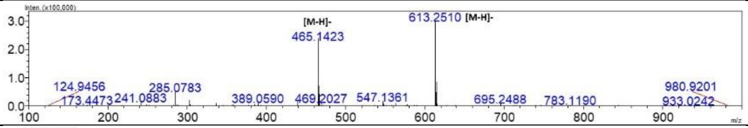


Peak 36

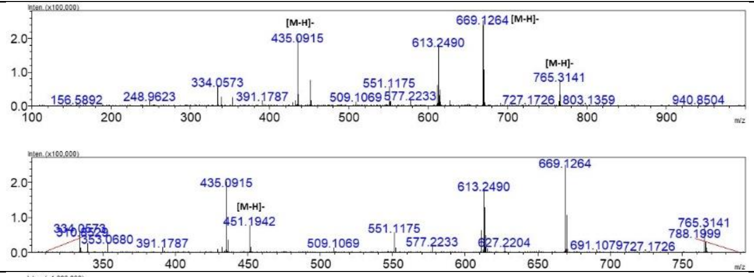


Peak 37

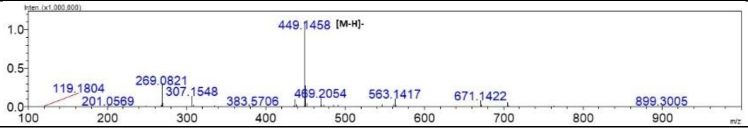

Peak 38

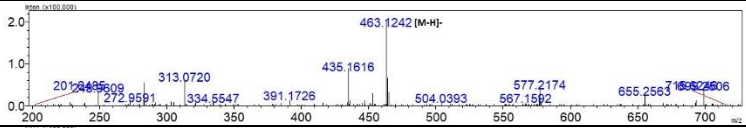


Peak 39

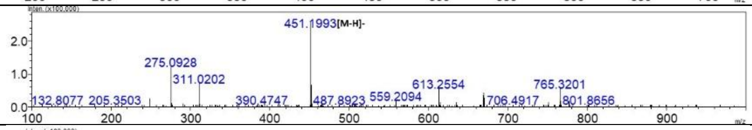


Peak 40

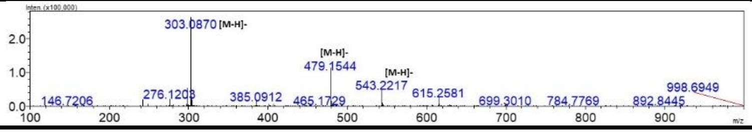

Supplement: Supplementary file 1 [file cells-11-00931-s001.zip › Table S3.pdf]
