# Supplementary material for: HPLC-PDA-ESI-HRMS-Based Profiling of Secondary Metabolites of Rindera graeca Anatomical and Hairy Roots Treated with Drought and Cold Stress
Source: Cells. 2022 Mar 8;11(6):931. doi: 10.3390/cells11060931 (PMC8946546; doi:10.3390/cells11060931)
Supplement: Supplementary file 1 [file cells-11-00931-s001.zip › Table S4.pdf]

HPLC-PDA-ESI-HRMS-Based Profiling of Secondary Metabolites of *Rindera graeca* Anatomical and Hairy Roots Treated With Drought And Cold Stress

Marcin R. Naliwajski [1#], Beata Wileńska[2,3*#], Aleksandra Misicka[2,3], Agnieszka Pietrosik[4] and Katarzyna Sykłowska-Baranek[4]

[1] Department of Plant Physiology and Biochemistry, Faculty of Biology and Environmental Protection, University of Lodz, 12/16 Banacha St., 90-237 Lodz, Poland; marcin.naliwajski@biol.uni.lodz.pl (M.R.N.)

[2] Faculty of Chemistry, University of Warsaw, 1 Pasteura St., 02-093 Warsaw, Poland; bwilenska@chem.uw.edu.pl (B.W.); misicka@chem.uw.edu.pl (A.M.)

[3] Biological and Chemical Research Centre, 101 Żwirki i Wigury St., 02-097 Warsaw, Poland

[4] Department of Pharmaceutical Biology and Medicinal Plant Biotechnology, Faculty of Pharmacy, Medical University of Warsaw, 1 Banacha St, 02-097 Warsaw, Poland; katarzyna.syklowska-baranek@wum.edu.pl (K.S.B.); agnieszka.pietrosiuk@wum.edu.pl (A.P.)

[#] These authors contributed equally to the work.

* Correspondence: bwilenska@chem.uw.edu.pl

Table S4. HRMS data for compounds detected in examined root extracts.

Extracts of RgAR root line					
**Peak No.**	**Tr**	**Molecular formula**	**Theoretical m/z**	**Measured m/z**	**Mass accuracy [ppm]**
1	19.39	C9H8O4	179.0350	179.0351	0.56
2	19.88	C15H22O8	329.1242	329.1229	3.95
		C15H18O9	341.0878	341.0867	3.22
3	21.22	C20H24N4O7	431.1572	431.1570	0.46
4	21.90	C18H16O9	375.0722	375.0716	1.60
5	23.46	C22H28O13	499.1457	499.1442	3.01
6	24.13	C33H18O6	509.1031	509.1047	3.14
		C27H22O13	553.0988	553.0979	1.63
7	24.89	C16H14O4	269.0819	269.0816	1.11
		C17H14O6	313.0718	313.0705	4.15
		C34H28O12	627.1508	627.1494	2.23
		C36H28O16	715.1305	715.1296	1.26
8	26.58	C54H22O4 (2-)	366.0686*	366.0681	1.37
9	27.24	C26H32N2O9	515.2036	515.2031	0.78
10	27.54	C25H31N3O4	436.2242	436.2242	0.00
11	27.99	C27H22O12	537.1038	537.1042	0.74
		C32H34O8	545.2181	545.2160	3.85
12	28.14	C28H32N16O18	439.0967	439.0966	0.23
13	28.29	C20H20O11	435.0933	435.0948	3.45
		C24H26O13	521.1301	521.1299	0.38
14	28.57	C18H16O8	359.0772	359.0782	2.78
		C21H18N6O or	369.1469	369.1460	2.44
		C20H22N2O5	369.1456	369.1460	1.08
		C22H28O12	483.1508	483.1513	1.03
15	29.55	C18H16O8	359.0772	359.0782	2.78
16	29.71	C22H24O10	447.1297	447.1286	2.46
		C22H22O11	461.1089	461.1108	4.12
		C28H36O16	627.1931	627.1933	0.32
17	30.11	C36H30O16	717.1461	717.1489	3.90
18	30.71	C21H24N2O5	383.1612	383.1624	3.13
		or C22H20N6O	383.1626	383.1624	0.52
19	30.83	C17H20O7	335.1136	335.1151	4.48
		C22H22O11	461.1089	461.1077	2.60
		C25H34O14	557.1876	557.1869	1.26
20	31.66	C28H24O12	551.1195	551.1214	3.45
21	33.63	C22H26O11	465.1402	465.1415	2.79
		C29H42O14	613.2502	613.2514	1.96
22	33.82	C29H40O14	611.2345	611.2323	3.60
		C35H24O14	669.1250	669.1242	1.20
		C48H46O9	765.3069	765.3102	4.31

23	34.33	C22H26O10	449.1453	449.1448	1.11
24	34.87	C23H32O9	451.1974	451.1958	3.55
25	37.99	C16H16O6	303.0874	303.0876	0.66
26	41.07	C13H12O3	215.0714	215.0721	3.25
**Extracts of RgTR7 root line**					
1	19.43	C9H8O4	179.0350	179.0348	1.12
2	19.78	C15H22O8	329.1243	329.1234	2.43
		C15H18O9	341.0878	341.0871	2.05
3	21.27	C19H28O11	431.1559	431.1566	1.62
4	21.90	C29H28O9	519.1661	519.1676	2.89
5	22.40	C15H12O5	271.0612	271.0623	4.06
		C16H10O6	297.0405	297.0413	2.69
		C14H12N4O4	299.0786	299.0792	2.01
		C18H24O11	415.1246	415.1257	2.65
		C25H30O14	553.1563	553.1550	2.35
6	22.69	C18H24O12	431.1195	431.1177	4.18
		C22H28O12	483.1508	483.1515	1.45
		C32H36O10	579.2236	579.2232	0.69
7	23.41	C16H22O10	373.1140	373.1140	0.00
		C18H24O10	399.1297	399.1290	1.75
		C23H24O11	475.1246	475.1247	0.21
		C24H24O13	519.1144	519.1136	1.54
8	23.90	C22H28O13	499.1457	499.1469	2.40
9	24.23	C16H18O8	337.0929	337.0938	2.67
		C15H32O16	467.1618	467.1602	3.42
		C26H22O11	509.1089	509.1069	1.37
		C27H22O13	553.0988	553.0996	1.45
10	24.57	C24H22O12	501.1039	501.1016	4.39
		C26H30O16	597.1461	597.1440	3.52
		C33H32O17	349.0747	349.0737	2.86
11	24.83	C16H14O4	269.0819	269.0828	3.34
		C17H14O6	313.0718	313.0706	3.83
		C34H28O12	627.1508	627.1511	0.48

		C30H28N2O16	671.1366	671.1372	0.89
		C36H28O16	715.1305	715.1301	0.56
12	25.70	C38H28O3	531.1966	531.1980	2.64
13	25.89	C22H30O13	501.1614	501.1615	0.20
14	26.13	C25H28N2O12	547.1569	547.1566	0.55
		C26H30O15	581.1512	581.1503	1.55
		C36H26O17	729.1097	729.1077	2.74
15	26.63	C36H30O17	733.1410	733.1389	2.86
16	27.26	C26H32N2O9	515.2035	515.2043	1.55
		C26H30O15	581.1512	581.1500	2.06
		C36H30O16	717.1461	717.1457	0.56
17	27.54	C25H31N3O4	436.2242	436.2240	0.46
		C22H24O12	479.1195	479.1191	0.83
18	28.01	C27H22O12	537.1038	537.1043	0.93
19	28.17	C20H28O12	459.1508	459.1515	1.52
		C38H36ON6O19	879.1962	879.1982	2.27
20	28.32	C20H20O11	435.0933	435.0935	0.46
21	28.57	C19H16O9	369.0722	369.0726	1.03
		C24H26O13	521.1301	521.1285	3.07
		C36H28O16 or	357.0616	357.0610	1.68
		C42H24O11	715.1246	715.1278	4.47
22	29.14	C20H20O11	435.0933	435.0928	1.15
23	29.65	C18H16O8	359.0772	359.0769	2.51
		C26H22O10	493.1140	493.1152	2.43
24	29.71	C22H24O10	447.1297	447.1288	2.01
		C28H36O16	627.1931	627.1951	3.19
25	30.30	C36H30O16	717.1461	717.1479	2.51
26	30.74	C21H24N2O5	383.1612	383.1606	1.57
27	30.83	C22H22O11	461.1089	461.1090	0.22
28	31.20	C22H22O10	445.1140	445.1145	1.12
29	31.71	C28H24O12	551.1195	551.1201	1.09
30	32.15	C26H22O10	493.1140	493.1136	0.81
		C31H30O15	641.1512	641.1507	0.78

31	32.64	C16H8O7	311.0197	311.0196	0.32
32	32.99	C38H36O9	635.2287	635.2259	4.41
33	33.66	C29H42O14	613.2502	613.2510	1.30
34	33.90	C23H32O9	451.1974	451.1971	0.66
		C41H50O14	765.3128	765.3137	1.18
35	34.36	C22H26O10	449.1453	449.1464	2.45
36	34.95	C15H16O5	275.0925	275.0914	4.00
		C17H14O6	313. 0718	313.0715	0.96
		C23H32O9	451.1974	451.1957	3.77
**Extracts of RgTR17 root line**					
1	19.58	C9H8O4	179.0350	179.0356	3.35
2	19.91	C15H22O8	329.1242	329.1229	3.95
		C15H18O9	341.0878	341.0867	3.22
3	21.10	C19H28O11	431.1559	431.1561	0.46
4	21.40	C22H28O13	499.1457	499.1434	4.61
5	21.97	C22H32O14	519.1719	519.1695	4.62
6	22.46	C15H12O5	271.0612	271.0604	2.95
		C25H30O14	553.1563	553.1556	1.27
7	22.72	C22H28O12	483.1508	483.1510	0.41
8	23.46	C26H20O7	443.1136	443.1157	4.74
9	23.96	C22H28O13	499.1457	499.1449	1.60
10	24.10	C26H22O11	509.1089	509.1082	1.37
		C27H22O13	553.0988	553.0986	0.36
11	24.58	C21H30O14	505.1563	505.1555	1.58
12	24.85	C16H14O4	269.0819	269.0822	1.11
		C17H14O6	313.0718	313.0708	3.19
		C34H28O12	627.1508	627.1503	0.80
		C36H28O16	715.1305	715.1293	1.68
13	25.95	C22H30O13	501.1614	501.1615	0.20
14	26.13	C12H12N4O	227.0938	227.0937	0.44
		C30H28O10	547.1610	547.1626	2.92
		C26H30O15	581.1512	581.1517	0.86
		C36H26O17	729.1097	729.1067	4.11
15	26.50	C36H30O17	733.1410	733.1386	3.27
16	26.77	C22H32O9	439.1974	439.1964	2.28
		C20H28O12	459.1508	459.1509	0.22
		C22H30O13	501.1614	501.1607	1.40
		C21H17NO7	534.1240	534.1243	0.56
17	27.23	C31H32O7	515.2075	515.2056	3.69
18	27.53	C25H31N3O4	436.2242	436.2245	0.69

19	27.93	C18H12O7	339.0510	339.0503	2.06
		C20H28O12	459.1508	459.1515	1.52
		C27H22O12	537.1038	537.1032	1.12
20	28.14	C60H32O8	439.0976	439.0969	1.59
21	28.30	C20H20O11	435.0933	435.0941	1.84
22	28.58	C18H16O8	359.0772	359.0767	1.39
		C22H28O12	483.1508	483.1508	0.00
		C36H28O16	715.1305	715.1291	1.96
23	29.16	C20H20O11	435.0933	435.0944	2.53
24	29.55	C18H16O8	359.0772	359.0763	2.51
		C26H22O10	493.1140	493.1140	0.00
		C27H22O12	537.1038	537.1036	0.37
25	29.72	C20H20O10	419.0984	419.0984	0.00
		C22H24O10	447.1297	447.1275	4.92
		C17H26N2O12	449.1413	449.1423	2.23
		C28H36O16	627.1931	627.1912	3.03
26	30.17	C36H30O16	717.1461	717.1464	0.42
27	30.74	C26H24O3	383.1653	383.1638	3.91
28	30.86	C17H20O7	335.1136	335.1150	4.18
		C22H26O11	465.1402	465.1413	2.36
		C16H34O17	497.1723	497.1717	1.21
		C29H26O10	533.1453	533.1452	0.19
		C25H34O14	557.1876	557.1891	2.69
		C27H32O16	611.1618	611.1594	3.93
29	31.26	C22H22O10	445.1140	445.1159	4.27
30	31.55	C23H24O11	475.1246	475.1257	2.32
		C35H30N2O14	701.1624	701.1629	0.71
31	31.69	C28H24O12	551.1195	551.1218	4.17
32	32.28	C31H30O15	320.0720	320.0720	0.00
		C27H44O19	335.1166	335.1160	1.79
		C24H28O12	507.1508	507.1500	1.58
		C25H32O13	539.1770	539.1758	2.23
		C31H30O15	641.1512	641.1519	1.09
33	32.41	C22H26O11	465.1402	465.1417	3.22
34	32.59	C22H30O19	453.1766	453.1763	0.88
35	33.67	C29H42O14	613.2502	613.2510	1.30
36	33.90	C17H12N10O5	435.0919	435.0915	0.92
		C19H28N6O7	451.1947	451.1942	1.11
		C36H26O14	669.1250	669.1264	2.09
		C41H50O14	765.3128	765.3141	1.70
37	34.36	C22H26O10	449.1453	449.1458	1.11
38	34.57	C22H24O11	463.1246	463.1242	0.86
39	34.91	C23H32O9	451.1974	451.1993	4.21
40	38.01	C16H16O6	303.0874	303.0870	1.32
		C23H28O11	479.1559	479.1544	3.13
		C29H36O10	543.2236	543.2217	3.50

* double charged ion
